# A computational dynamics and damping–induced transitions in beam–foundation systems with viscoelastic constraints

**DOI:** 10.1371/journal.pone.0359573

**Published:** 2026-10-07

**Authors:** Muna Elsadig, Shamsa Kanwal, Rab Nawaz, Qazi Muhammad Zaigham Zia

**Affiliations:** 1 Department of Information Systems, College of Computer and Information Sciences, Princess Nourah bint Abdulrahman University, Riyadh, Saudi Arabia; 2 Department of Mathematics, COMSATS University Islamabad, Tarlai Kalan, Islamabad, Pakistan; 3 Center for Applied Mathematics and Bioinformatics (CAMB), Gulf University for Science and Technology, Hawally, Kuwait; Universiti Teknologi Malaysia, MALAYSIA

## Abstract

This study examines the vibrational dynamics of elastically constrained beam foundation systems by considering Timoshenko, shear, Rayleigh, and Euler–Bernoulli beam models resting on viscoelastic Filonenko-Borodich and Hetényi foundations. Emphasis is placed on understanding how damping, foundation stiffness, shear deformation, and rotary inertia govern dynamic transitions across undamped, underdamped, and overdamped regimes. Using separation of variables for the analytical solution and the Galerkin finite element method for the numerical solution, non–classical boundary conditions are used for the formulation of the equations. Analytical and numerical solutions are employed to explore frequency characteristics, oscillation amplitudes, and stability behavior. The results reveal that increasing foundation stiffness enhances natural frequencies and contributes to effective vibration control, while increasing damping induces a gradual transition from sustained oscillations to complete vibration suppression. These damping-driven transitions highlight complex dynamic behavior relevant to nonlinear systems theory. Excellent agreement between analytical and numerical findings confirms the robustness of the proposed formulation. The study underscores the critical role of viscoelastic foundations and damping mechanisms in shaping nonlinear vibration responses, offering insights applicable to the design of advanced structural systems. Extensions to non–uniform geometries, heterogeneous materials, and environmental effects are expected to further enrich the dynamical behavior and practical relevance of the model.

## Introduction

Beam vibrational behavior has major implications for structural and mechanical engineering. Every structural configuration is dependent on the support given by beams, and its relevance goes beyond technical domains. Beams are commonly used in civil engineering to support steel frames, buildings, and bridges. Entire buildings are simplified into beams, making early modeling easier. In mechanical engineering, beam constructions include shaft systems with pulleys and gears, as well as machine frames mounted on trucks. Robotic arms used in industrial operations are likewise classified as beam structures.In aerospace engineering, both straight and curved beam structures are used in the design of aircraft and spacecraft. Preliminary assessments sometimes simplify these components, such as an airplane’s wings, into beam structures. Given their widespread use in engineering, a thorough understanding of beam response, as well as informed material and design decisions, is critical to ensuring the longevity, safety, and functionality of the systems that rely on them. Different beam models are used to investigate the behavior of beams in diverse settings. The Euler–Bernoulli beam model is used to study narrow beams, while the Timoshenko beam model is better suited to shorter and thicker beams. Shear beam theory is often used for composite beams, and Rayleigh beam theory improves the Euler–Bernoulli beam model by incorporating the influence of rotating inertia, providing greater accuracy in specific conditions. The structure and nature of the applied loads determine which beam model is most appropriate. Each theory has limitations, and understanding them is critical for conducting accurate and reliable analysis.

Furthermore, in beam dynamics, vibrations in beams resting on deformable surfaces, known as elastic foundations, are an important feature of structural analysis. Soil, concrete slabs, and other flexible materials can all be used as foundations. When a beam is subjected to stresses or intrinsic excitations, it may vibrate, which affects the foundation beneath it. The study of beam vibrations on elastic foundations is very essential in structural engineering since it affects the performance and stability of various structures. Elastic foundations are used in many mechanical and civil systems to provide support and vibration isolation. Several foundation models, such as Winkler, Pasternak, Vlasov, and Filonenko–Borodich, are used to represent different types of elastic support along a beam’s axis.

Bresse proposed a beam theory in the mid–1800s that includes the shear effects and bending of a straight beam via two different kinematic fields, namely the rotation and displacement variables. Bresse also examined the spinning impact independently. In the early 1900s, Timoshenko expanded Bresse’s theory by incorporating shear correction into fully consistent beam construction [1,2], and [3]. Problems with vibrating beams without elastic support have been carefully investigated. Chun [4] examines the beam problem with one end supported and the other end unsupported. Lee [5] solves the characteristic equation of a beam with a rotating spring and mass at one end. Lai et al. [6] investigates beam dynamics using the Adomian decomposition approach. Smith et al. [7] successfully addressed fixed beam boundary issues using the sinc–Galerkin approach. Abbas [8] investigated the behavior of Timoshenko beams under non–classical boundary conditions. Abbas and Thomas [9] significantly contributed to our understanding of higher–mode dynamics in Timoshenko beams. Their findings underscored the importance of including both rotational inertia and shear deformation in frequency analysis, as well as the limitations of traditional beam theories in adequately characterizing the vibrational behavior of shear–deformable beams. Abbas also researched TB vibration under elastic constraints. Rao [10] investigated a clamped–clamped beam with intermediary support. Oni and Zhou [11,12] have thoroughly researched and detailed the impacts of elastic foundations in the literature. Adhikari [13] conducted a thorough examination into the analysis and identification of multi–parameter damped mechanical systems, focusing on viscous and non–viscous damping in linear multi–degree–of–freedom vibrations. His research provided a solid theoretical foundation for simulating damping in complex structural dynamics. In [14–19], the authors investigated the interaction of structure with various types of foundations with extraordinary attention to detail. Hsu [16], Shin et al. [20], and Rosa [21] investigated the reaction of a beam to a single parametric elastic Winkler foundation. Hetenyi [22] deals with the uniform Euler–Bernoulli beams supported by the Winkler foundation. In their foundational work, Pavlovic and Doyle [23] carried out a vibrational analysis of beams resting on elastic partial foundations. Kacar et al. [24] applied the differential transform approach to study the dynamics response of a beam resting on an elastic Winkler foundation. The Hetenyi and the Pasternak foundations, recognized as two–parametric models, have transverse shear modulus or shear rigidity and flexural rigidity, respectively. In two parametric foundation models, the initial parameter of the foundation is still considered to be the elastic Winkler foundation parameter. The studies of Shin et al. [20], Arboleda–Monsalve et al. [25], Zhu [26], and Civalek [27] were focused on the Pasternak foundations. Wang and Stephens [28] observed the eigenfrequencies of a Timoshenko beam over a Pasternak foundation, and in the same work, they calculated characteristic equations for beams with different boundary conditions. The dynamic behavior of a damped beam with elastic supports was studied by Panigrahi and Mahapatra [29], who utilized a Fourier cosine series. Zhao and Chang [30] explored the forced and free vibrations of a double beam with arbitrary end conditions that are connected to a viscoelastic layer and to discrete points. By employing stress–based FEM, Wieckowski and Swiatkiewicz [31] study to solve the static bending problem for Euler–Bernoulli and Timoshenko beams.

Several studies have emphasized the importance of nonlinear effects, damping, analytical approximation, and stability analysis in dynamical and vibrational systems. A nonlinear oscillatory system with viscous damping has been studied using the multiple–scales method, with analytical solutions validated by fourth–order Runge–Kutta simulations (RK4) and stability characteristics analyzed by bifurcation and resonance analysis [32]. Likewise, analytical approximation techniques based on Laplace transforms and homotopy perturbation have been applied to obtain bound solutions to nonlinear Duffing–type equations, with numerical and qualitative validation provided by RK4 calculations and linearized stability analysis [33]. Multiple–scales methods have also been applied to the dynamics of coupled damped oscillatory systems, with special attention paid to resonance conditions, steady–state responses, and stability regions. In rigid–body dynamics and spacecraft dynamics under external torques, gyrostatic effects, electromagnetic interactions, and resistive environments, damping and stability considerations have been explored beyond oscillator models. In engineering dynamical systems, dissipative and stability mechanisms have broader significance studied by a number of investigations including [34–36], and [37]. It has been shown in these studies that damping and stability considerations are important in vibration analyses; however, the combined impact of damping, beam–theory effects, two–parameter elastic foundations, and non–classical elastic boundary conditions remains underexplored. Using analytical and numerical approaches, G. Kanwal et al. [38] presented an analysis of the damped vibrational response of beams on multi–parametric foundations, focusing particularly on the influence of foundation parameters. The present study investigates four beam theories, namely Euler–Bernoulli, shear, Rayleigh, and Timoshenko beams, resting on two–parameter Hetényi and Filonenko–Borodich foundations. Furthermore, non–classical elastic boundary conditions involving translational and rotational springs are incorporated to represent partially restrained beam configurations. An integrated analysis of shear deformation, rotary inertia, foundation stiffness, elastic end restraints, and viscous damping is presented. Thus, the present study provides a unified comparison of four beam theories under elastic constraints and two–parameter foundation models.

Although the dynamic behavior of beams has long been a key focus of structural dynamics study, the combined influence of damping, elastic supports, and non–classical boundary conditions is frequently overlooked. Much earlier research has limited their assessments to classical boundary conditions, ignoring actual dampening effects that are inherent in real structural systems. This exclusion results in models that lack the stability required for accurate predictions in engineering applications involving elastic constraint and energy dissipation. Several numerical studies have explored the impact of rotating inertia and shear deformation on beams lying on Winkler and Pasternak foundations, however most of them assume conventional limitations or only consider one beam model [17]. The current paper bridges this research gap by providing a detailed evaluation of the free vibrational response of shear, Timoshenko, Rayleigh, and Euler–Bernoulli beams subjected to elastic restrictions and resting on foundation models such as the Filonenko and Hetényi foundations. The work distinguishes itself by using non–classical boundary conditions and employing both linear and rotational springs to better mimic actual structural constraints. Furthermore, it explicitly incorporates damping effects, as well as shear deformation, rotary inertia, and foundation stiffness characteristics, all of which have a significant impact on beam dynamics. The Galerkin finite element method and the separation of variables methodology are used in this study to solve the complex couple equations. This methodological technique not only avoids shear locking, but also provides speedy convergence that closely matches precise analytical results. Extensive research focuses on undamped, underdamped, and overdamped frequencies, offering a nuanced understanding of how damping interacts with non–classical limitations and elastic supports. The results show that the addition of shear layers, flexural stiffness, foundation constants, and a damping effect significantly increases the natural frequencies and changes the vibrational properties of the beams. The combined effects of shear deformation, rotating inertia, and damping create larger variations in the dynamic response than any single element alone, especially for Euler–Bernoulli beams, which are generally thought to ignore such interactions. Unlike commercial software such as ANSYS, which frequently simplifies or ignore the combined effect of damping, non–classical boundaries, and elastic supports. The proposed approach allows for accurate modeling of complex real–world circumstances. This capability improves predictive accuracy for real applications such as pipelines, railway tracks, mechanical systems, and microscale devices, all of which require precise vibration modeling to ensure performance, longevity, and safety. Overall, this paper bridges a critical research gap by providing a solid framework for advanced beam theories, non–classical boundary conditions, elastic foundations, and damping effects. Finally, these findings expand our understanding of structural dynamics and pave the path for the construction of more accurate analytical and numerical models, thereby making modern engineering techniques safer and more efficient.

The article begins by presenting the problem formulation, followed by the analytical determination of the undamped and damped frequencies of a beam connected to rotational and translational springs. The corresponding frequencies are also evaluated using the finite element method. The results obtained from both approaches are then presented and discussed, followed by the main conclusions of the study.

## Statement of the problem

The problem concerns the transverse vibrational behavior of a Timoshenko beam. This beam considers both rotary inertia and shear deformation, two aspects overlooked in the classic Euler–Bernoulli beam theory, where cross–sections are perpendicular to the neutral axis (refer to Figs 1-Fig 2). In contrast, the Timoshenko beam permits these cross-sections to rotate, which is a more refined presentation of the deformation. The beam is elastically supported at the ends and placed on elastic foundations, which are described as the Hetényi and Filonenko models in (1) and (2). To derive natural frequencies and corresponding mode shapes, the beam’s dynamics must be analyzed for several elastic foundations and solved using the coupled system of differential equations.


$$\begin{array}{c} \rho A\left(\frac{\partial^{2}z(x,t)}{\partial t^{2}}\right)-KGA\left(\frac{\partial^{2}z(x,t)}{\partial x^{2}}-\frac{\partial \psi }{\partial x}\right)+K_{h}z(x,t)-q_{f}\left(\frac{\partial^{2}z(x,t)}{\partial x^{2}}\right)+F_{D}\frac{\partial z(x,t)}{\partial t}=0 \end{array}$$ (1a)

$$\begin{array}{c} \rho I\left(\frac{\partial^{2}\psi }{\partial t^{2}}\right)-EI\left(\frac{\partial^{2}\psi }{\partial x^{2}}\right)-KGA\left(\frac{\partial z(x,t)}{\partial x}-\psi \right)=0 \end{array}$$ (1b)

**Fig 1:**
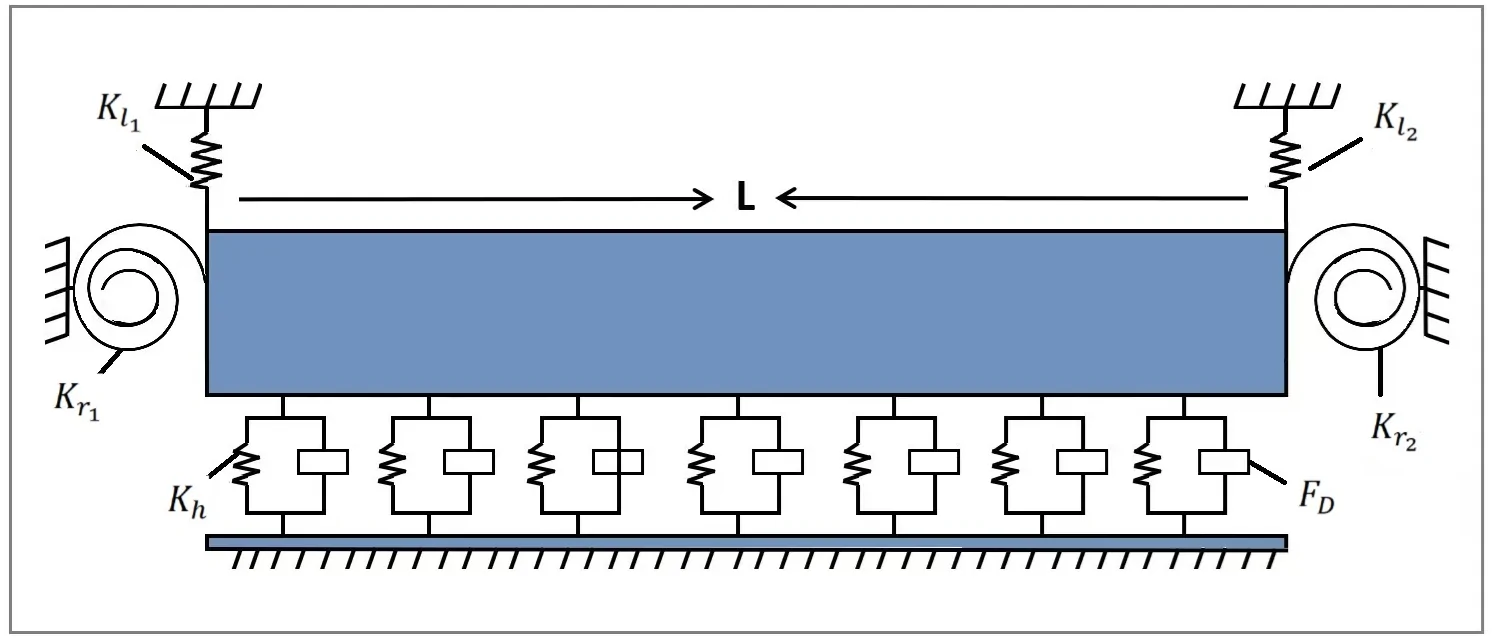
Timoshenko model resting over Hetényi foundation.

**Fig 2:**
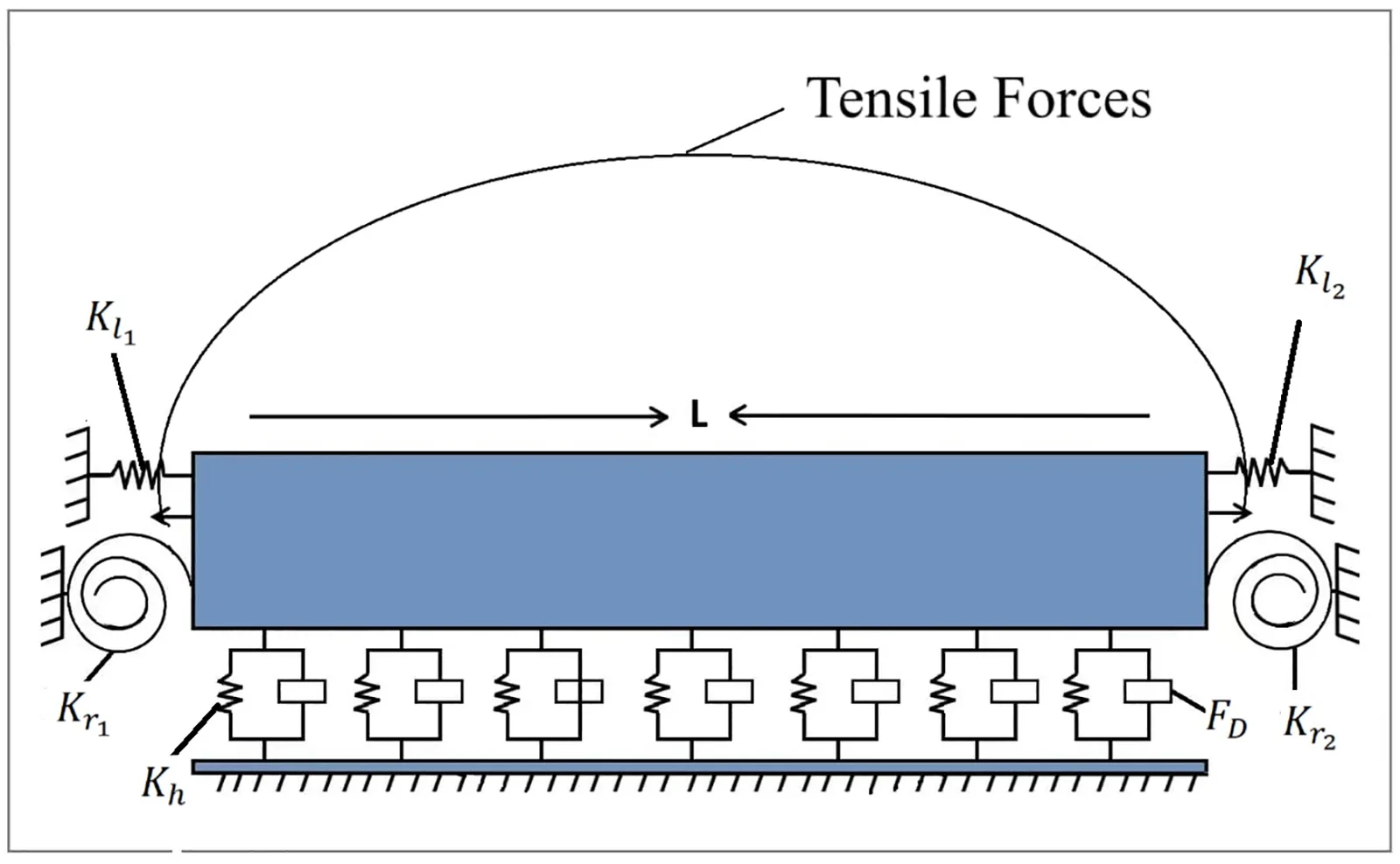
Timoshenko model resting over Filonenko-Borodich foundation.

The Winkler foundation constant modulus of rigidity, shear modulus, bending slope, and cross-section shape factors are represented by $K_{h}$ (N/m^2^), *G* (Pa), ψ (rad), and *K* (dimensionless), respectively. And *E* (Pa), ρ (kg/m^3^), *A* (m^2^), *I* (m^4^), and *z*(*x*, *t*) (m) are the Young’s modulus of the beam, mass density, cross-sectional area, second moment of inertia, and displacement, respectively. Finally, the damping modulus ($F_{D}$, N·s/m) describes the viscous damping a*tt*ributes of the medium in the given model. The translational and rotational spring constants, $K_{l_{1}},K_{l_{2}}$ (N/m) and $K_{r_{1}},K_{r_{2}}$ (N·m/rad), represent the elastic end restraints of the beam. Usually, for a two-dimensional Filonenko elastic foundation, $q_{f}=T_{s}$ (N), the shear-layer tension parameter, while $q_{f}=EI$ (N·m^2^) for the Hetényi elastic foundation, where *EI* is the flexural rigidity. Note that $q_{f}$ therefore carries different physical dimensions depending on the foundation type: a force (N) for the Filonenko–Borodich case and a flexural rigidity (N·m^2^) for the Hetényi case; the governing equation (2.1a) is applied consistently to each case with $q_{f}$ interpreted according to the corresponding foundation model. If $q_{f}=0$ is assumed, then it means the governing equation for a Timoshenko beam is on a Winkler foundation.

Since the partial differential equations for β and *z* (*x*, *t*) can only be decoupled when the cross-sectional area and density do no*t* vary, they are identical. The elastically constrained Timoshenko beam has the following pertinent boundary conditions:


$$\begin{array}{cc} -KGA\left(\frac{\partial z(0,t)}{\partial x}-\psi \right) & =-K_{l_{1}}z(0,t)\;\;\;\;\; \end{array}$$ (2a)

$$\begin{array}{cc} -KGA\left(\frac{\partial z(L,t)}{\partial x}-\psi \right) & =K_{l_{2}}z(L,t)\;\;\;\;\; \end{array}$$ (2b)

$$\begin{array}{cc} EI\frac{\partial \psi (0,t)}{\partial x} & =K_{r_{1}}\psi (0,t)\;\;\;\;\; \end{array}$$ (2c)

$$\begin{array}{cc} EI\frac{\partial \psi (L,t)}{\partial x} & =-K_{r_{2}}\psi (L,t)\;\;\;\;\; \end{array}$$ (2d)

Here, the rotational and translational spring constants at the left and right ends of Timoshenko beams are denoted by $K_{r_{1}}$, $K_{r_{2}}$, $K_{l_{1}}$, and $K_{l_{2}}$, respectively. The system’s initial conditions are enumerated below:


$$\begin{array}{c} z(x,0)=0.1\;\text{and}\;\frac{\partial z(x,t)}{\partial t}=0 \end{array}$$

Remarkably, the Rayleigh beam, Euler-Bernoulli, and shear beams can be acquired as special cases when the rotary inertia is eliminated from Eqs. (1a-1b) The equations refer to a shear beam supported by two-parametric foundations. And by eliminating the shear deformation effect, Eqs. (1a-1b) result in the Rayleigh beam. Once shear deformation and rotary inertia effects have been omitted, Eqs. (1a-1b) become the Euler-Bernoulli beam model, which incorporates elastic foundations. Additionally, the boundary conditions reduce to the classical boundary conditions for appropriate limiting values of the spring constants. The numerical and analytical approaches are then employed to determine the eigenfrequencies and corresponding eigenmodes.

## Analytical formulation

The method of separation of variables is utilized for the solution of the coupled differential equations (1a-1b). which refers to the fact that, the time solution *T*(*t*) should be separable from the spatial solutions for the displacement and bending slope of the beam, i.e., the bending slope and the displacement of the beam are only dependent on position. Additionally, the synchronization of the time for the beam displacement and bending slope must ensure that these two vary together, thereby giving a more physically realistic model.


$$\begin{array}{c} \left[\begin{array}{c} z\left(x,t\right)\\ \psi \left(x,t\right) \end{array}\right]=T(t)\left[\begin{array}{c} X\left(x\right)\\ \eta \left(x\right) \end{array}\right] \end{array}$$ (3)

The Eq. (3) is inserted into Eqs. (1a–1b) yields:


$$\begin{array}{c} \rho AXT^{{\;}^{\prime \prime }}-(KGAX^{{\;}^{\prime \prime }}-KGA\eta^{{\;}^{\prime }}-K_{h}X+q_{f}X^{{\;}^{\prime \prime }})T+F_{D}XT^{{\;}^{\prime }}=0 \end{array}$$ (4a)

$$\begin{array}{c} EI\eta^{{\;}^{\prime \prime }}T+KGA(X^{{\;}^{\prime }}-\eta )T+\rho IXT^{{\;}^{\prime \prime }}=0 \end{array}$$ (4b)

The prior equations are divided by the ηT and *XT*, respectively. The Eqs (4a) and (4b) could be written as:


$$\begin{array}{c} \frac{T^{{\;}^{\prime \prime }}}{T}+\frac{F_{D}T^{{\;}^{\prime }}}{T}=\frac{KGX^{{\;}^{\prime \prime }}}{\rho X}+\frac{KG\eta^{{\;}^{\prime }}}{\rho X}+\frac{K_{h}}{\rho A}-\frac{q_{f}X^{{\;}^{\prime \prime }}}{\rho AX} \end{array}$$ (5a)

$$\begin{array}{c} \frac{T^{{\;}^{\prime \prime }}}{T}=-\frac{E\eta^{{\;}^{\prime \prime }}}{\rho \eta }-\frac{KGAX^{{\;}^{\prime }}}{\rho I\eta }+\frac{KGA}{\rho I} \end{array}$$ (5b)

Owing to the separation of the variables, both sides of Eqs. (5a) and (5b) should be equal to a constant, say $-\omega^{2}$. Consequently, we will write:


$$\begin{array}{c} \rho AT^{{\;}^{\prime \prime }}+F_{D}T^{{\;}^{\prime }}+\rho A\omega^{2}T=0\\ X^{{\;}^{\prime \prime }}(KGA+q_{h})-KGA\eta^{{\;}^{\prime }}-K_{h}X+\omega^{2}\rho AX=0 \end{array}$$ (6)

$$\begin{array}{c} T^{{\;}^{\prime \prime }}+\omega^{2}T=0\\ EI\eta {\;}^{\prime \prime }+KGA(X^{{\;}^{\prime }}-\eta )+\omega^{2}\rho I\eta =0 \end{array}$$ (7)

The following equation can be used to represent the foremost spatial equations from (6–7).


$$\begin{array}{c} (\rho I+\rho A)T^{{\;}^{\prime \prime }}+F_{D}T^{{\;}^{\prime }}+(\rho A+\rho I)\omega^{2}T=0 \end{array}$$ (8)

And the second spatial equation from Eqs. (6–7) can be written in the matrix notation as follows:


$$\begin{array}{cc} & \left[\begin{array}{cc} KGA+q_{f} & 0\\ 0 & EI \end{array}\right]\left[\begin{array}{c} X^{{\;}^{\prime \prime }}\left(x\right)\\ \eta^{{\;}^{\prime \prime }}\left(x\right) \end{array}\right]+\left[\begin{array}{cc} 0 & -KGA\\ KGA & 0 \end{array}\right]\left[\begin{array}{c} X^{{\;}^{\prime }}\left(x\right)\\ \eta^{{\;}^{\prime }}\left(x\right) \end{array}\right]\\ & +\left[\begin{array}{cc} \omega^{2}\rho A-K_{h} & 0\\ 0 & -KGA+\omega^{2}\rho I \end{array}\right]\left[\begin{array}{c} X\left(x\right)\\ \eta \left(x\right) \end{array}\right]=0 \end{array}$$ (9)

Decoupling of the above equations will yield


$$\begin{array}{cc} & EIX^{iv}+\frac{EIq_{f}X^{iv}}{KGA}-q_{f}X^{{\;}^{\prime \prime }}+K_{h}X-\frac{K_{h}EIX^{{\;}^{\prime \prime }}}{KGA}-\omega^{2}\rho AX+\frac{\omega^{2}\rho AEIX^{{\;}^{\prime \prime }}}{KGA}+\frac{\omega^{2}\rho Iq_{f}X^{{\;}^{\prime \prime }}}{KGA}+\\ & \frac{\omega^{2}K_{h}\rho IX}{KGA}-\frac{\omega^{4}\rho^{2}AIX}{KGA}+\frac{\omega^{2}\rho IX}{KGA}=0\\ & EI\eta^{iv}+\frac{EIq_{f}\eta^{iv}}{KGA}-q_{f}\eta^{{\;}^{\prime \prime }}+K_{h}\eta -\frac{K_{h}EI\eta^{{\;}^{\prime \prime }}}{KGA}-\omega^{2}\rho A\eta +\frac{\omega^{2}\rho AEI\eta^{{\;}^{\prime \prime }}}{KGA}+\frac{\omega^{2}\rho Iq_{f}\eta^{{\;}^{\prime \prime }}}{KGA}+\\ & \frac{\omega^{2}K_{h}\rho I\eta }{KGA}-\frac{\omega^{4}\rho^{2}AI\eta }{KGA}+\frac{\omega^{2}\rho I\eta }{KGA}=0 \end{array}$$ (10)

The above fourth-order differential equations for η and *X* are found to have comparable forms, demonstrating that the solution to Eq. (10) can be obtained as a constant multiple.


$$\begin{array}{c} \left[\begin{array}{c} X\left(x\right)\\ \eta \left(x\right) \end{array}\right]=d\text{V}e^{rx} \end{array}$$ (11)

Here **V**, *r*, and *d* are eigenvectors, eigenvalues, and constant numbers. Subsequently, Eq. (11) will be utilized in Eq. (9), to obtain


$$\begin{array}{c} \left[\begin{array}{cc} KGAr^{2}+\rho \;A\omega^{2}+q_{f}r^{2}-K_{h} & -KGAr\\ KGAr & IEr^{2}-KGA+\omega^{2}\rho I \end{array}\right]\text{V}=0 \end{array}$$ (12)

By keeping the determinant of a matrix identical to 0, eigenvalues and eigenvectors are found using Eq. (12). So, we obtain


$$\begin{array}{c} r^{4}+\frac{r^{2}}{(KGAEI+q_{f}EI{)}^{2}}\left(-KGAq_{f}+\rho A\omega^{2}EI-K_{h}EIKGA+KGA\omega^{2}\rho I+q_{f}\rho I\omega^{2}\right)+\\ \frac{1}{KGAEI+q_{f}EI}\left(-KGA^{2}\rho \omega^{2}+\omega^{4}\rho^{2}AI+K_{h}KGA-K_{h}\omega^{2}\rho I\right)=0 \end{array}$$ (13)

As a result, the eigenvalues can be obtained from Eq. (14):


$$\begin{array}{c} r_{i}^{2}=\pm (E_{1}+E_{2})+\sqrt{(E_{1}-E_{2}{)}^{2}+E_{3}}\;\;\;i=1,\;2,\;3,\;4 \end{array}$$ (14)

where


$$\begin{array}{cc} & E_{1}=\frac{KGA\omega^{2}\rho I-KGAq_{f}+q_{f}\omega \rho I-K_{h}EI}{2(KGAEI+q_{f}EI)},\;\;E_{2}=\frac{EIA\rho \omega^{2}}{KGAEI+q_{f}EI},\\ & E_{3}=\frac{1}{(KGAEI+q_{f}EI{)}^{2}}(K^{2}G^{2}A^{3}\rho EI\omega^{2}-K_{h}K^{2}G^{2}A^{2}EI-K_{h}q_{f}KGAEI+\\ & K_{h}\omega^{2}\rho I^{2}EIKGA-K_{h}q_{f}EI^{2}\omega^{2}\rho -K_{h}E^{2}I^{2}\omega^{2}\rho A) \end{array}$$ (15)

And an associated eigenvector $V_{i}$ is formulated in Eq. (16):


$$\begin{array}{c} V_{i}=\left[\begin{array}{c} KGAr_{i}\\ KGA{r_{i}}^{2}+\rho \;A\omega^{2}+q_{f}{r_{i}}^{2}-K_{h} \end{array}\right]\;\;\;or\;\;\;\left[\begin{array}{c} EIr_{i}^{2}-KGA+\rho I\omega^{2}\\ -KGAr_{i} \end{array}\right] \end{array}$$ (16)

The spatial solution of the Eq. (13) is stated as


$$\begin{array}{cc} \left[\begin{array}{c} X\left(x\right)\\ \eta \left(x\right) \end{array}\right] & =\sum_{i=1}^{4}d_{i}V_{i}e^{r_{i}x}\\ & =d_{1}V_{1}e^{\beta x}+d_{2}V_{2}e^{-\beta x}+d_{3}V_{3}e^{i\alpha x}+d_{4}V_{4}e^{-i\alpha x} \end{array}$$ (17)

And here


$$\begin{array}{c} \alpha^{2}=(E_{1}+E_{2})+\sqrt{(E_{1}-E_{1}{)}^{2}+E_{3}},\;\;\beta^{2}=-(E_{1}+E_{2})+\sqrt{(E_{1}-E_{1}{)}^{2}+E_{3}} \end{array}$$

Eq. (17) can be reconstructed as follows by using hyperbolic and sinusoidal functions:


$$\begin{array}{c} \left[\begin{array}{c} X\\ \eta \end{array}\right]=\left[\begin{array}{c} A_{1}\\ B_{1} \end{array}\right]sin(\alpha x)+\left[\begin{array}{c} A_{2}\\ B_{2} \end{array}\right]cos(\alpha x)+\left[\begin{array}{c} A_{3}\\ B_{3} \end{array}\right]sinh(\beta x)+\left[\begin{array}{c} A_{4}\\ B_{4} \end{array}\right]cosh(\beta x) \end{array}$$ (18)

*A*_1_, *A*_2_, *A*_3_, *A*_4_, *B*_1_, *B*_2_, *B*_3_, and *B*_4_ are constants. The 8 constants in Eq. (18) appear to be undefined, so from Eq. (19) we can relate $A_{i}$ and $B_{i}$:


$$\begin{array}{cc} B_{1} & =\frac{-\alpha^{2}(KGA+q_{f})+(-K_{h}+\rho A\omega^{2})}{KGA\alpha }A_{2}\\ B_{2} & =\frac{-\alpha^{2}(KGA+q_{f})+(-K_{h}+\rho A\omega^{2})}{-KGA\alpha }A_{1}\\ B_{3} & =\frac{\beta^{2}(KGA+q_{f})+(-K_{h}+\rho A\omega^{2})}{KGA\beta }A_{4}\\ B_{4} & =\frac{\beta^{2}(KGA+q_{f})+(-K_{h}+\rho A\omega^{2})}{KGA\beta }A_{3} \end{array}$$ (19)

Thereafter, Eq. (18) has four unknowns to solve for. These relations can be gained more efficiently by putting the assumed solution (18) to the differential equations (9).

By using Eq. (18) in Eq. (2a-2d), we get the following:


$$\begin{array}{cc} & A_{1}(\alpha -(\frac{-\frac{\gamma^{2}\alpha^{2}q_{f}}{AGK}-\frac{\gamma^{2}K_{h}}{AGK}-\frac{\gamma^{2}q_{f}}{EI}+\gamma^{2}\beta^{2}Z+\alpha^{2}Z}{\alpha (\gamma^{2}+Z)}+\frac{\alpha q_{f}}{AGK}+\frac{K_{h}}{\alpha (AGK)}))-\\ & A_{2}(\frac{K_{l_{1}}}{AGK})+A_{3}(\beta -(\frac{-\frac{\gamma^{2}\beta^{2}q_{f}}{AGK}+\frac{\gamma^{2}K_{h}}{AGK}+\frac{\gamma^{2}q_{f}}{EI}+\gamma^{2}\alpha^{2}+\beta^{2}Z}{q(\gamma^{2}+Z)}+\frac{\beta q_{f}}{AGK}-\frac{K_{h}}{\beta (AGK\;)}))-\\ & A_{4}\frac{K_{l_{1}}}{AGK}=0 \end{array}$$ (20)

$$\begin{array}{cc} & A_{1}(-\frac{K_{r_{1}}}{EI}(\frac{-\frac{\gamma^{2}\alpha^{2}q_{f}}{AGK}-\;\frac{\gamma^{2}K_{h}}{AGK}-\frac{\gamma^{2}q_{f}}{+}\gamma^{2}\beta^{2}Z+\alpha^{2}Z}{\left(\alpha (\gamma^{2}+Z\right)}+\frac{\alpha q_{f}}{AGK}+\frac{K_{h}}{\alpha (AGK)}))EI)+\\ & A_{2}(\frac{\frac{\gamma^{2}\alpha^{2}q_{f}}{AGK}+\frac{\gamma^{2}K_{h}}{AGK}EI+\frac{\gamma^{2}\;q_{f}}{EI}-\gamma^{2}\beta^{2}Z-\alpha^{2}Z}{\gamma^{2}+Z}-\frac{\alpha^{2}q_{f}}{AGK}-\frac{K_{h}}{AGK})+\\ & A_{3}(-\frac{K_{r_{1}}}{EI}(\frac{-\frac{\gamma^{2}\beta^{2}q_{f}}{AGK}+\frac{\gamma^{2}K_{h}}{AGK}+\frac{\gamma^{2}q_{f}}{EI}+\gamma^{2}\alpha^{2}+\beta^{2}Z}{\beta (\gamma^{2}+Z)}+\frac{\beta q_{f}}{AGK}-\frac{K_{h}}{\beta (AGK)}))-\\ & A_{4}(\frac{-\frac{\gamma^{2}\beta^{2}q_{f}}{AGK}+\frac{\gamma^{2}K_{h}}{AGK}+\frac{\gamma^{2}q_{f}}{EI}+\gamma^{2}\alpha^{2}Z+\beta^{2}Z}{\gamma^{2}+Z}+\frac{\zeta^{2}q_{f}}{AGK}-\frac{K_{h}}{AGK})=0 \end{array}$$ (21)

$$\begin{array}{cc} & A_{1}(\cos(\alpha L)(\alpha -(\frac{-\frac{\gamma^{2}\alpha^{2}q_{f}}{AGK}-\frac{\gamma^{2}K_{h}}{AGK}-\frac{\gamma^{2}q_{f}}{EI}+\gamma^{2}\beta^{2}Z+\alpha^{2}Z}{\alpha (\gamma^{2}+Z)}+\frac{\alpha q_{h}}{AGK}+\frac{K_{h}}{\alpha (AGK)}))+\\ & \frac{K_{l_{2}}\sin(\alpha L)}{AGK})+A_{2}(\sin(\alpha L)(-(\frac{\frac{\gamma^{2}\alpha^{2}r}{AGK}+\frac{\gamma^{2}\;K_{h}}{AGK}+\frac{\gamma^{2}r}{EI}-\gamma^{2}\beta^{2}Z-\alpha^{2}Z}{\alpha (\gamma^{2}+Z)}-\frac{\alpha q_{f}}{AGK}-\frac{K_{h}}{\alpha (AGK)})\\ & -\alpha )+\frac{K_{l_{2}}\cos(\alpha L)}{AGK})+A_{3}(\cosh(\beta L)(\beta -(\frac{-\frac{\gamma^{2}\beta^{2}q_{f}}{AGK}+\frac{\gamma^{2}K_{h}}{AGK}+\frac{\gamma^{2}q_{f}}{EI}+\gamma^{2}\alpha^{2}Z+\beta^{2}Z}{q(\gamma^{2}+Z)}+\frac{\beta q_{f}}{AGK}-\\ & \frac{K_{h}}{\beta (AGK)}\;))+\frac{K_{l_{2}}\sinh(\beta L)}{AGK})+A_{4}((\sinh(\beta L)(\beta -(\frac{-\frac{\gamma^{2}\beta^{2}q_{f}}{AGK}+\frac{\gamma^{2}K_{h}}{AGK}+\frac{\gamma^{2}q_{f}}{EI}+\gamma^{2}p^{2}Z+q_{f}^{2}Z}{q(\gamma^{2}+Z)}+\\ & \frac{q_{f}}{AGK}-\frac{K_{h}}{q_{f}(AGK)}\;))+\frac{K_{l_{2}}\cosh(\beta L)}{AGK}))=0 \end{array}$$ (22)

$$\begin{array}{cc} & A_{1}(\frac{1}{EI}((K_{r_{2}}\cos(\alpha L)(\frac{-\frac{\gamma^{2}\alpha^{2}q_{f}}{AGK}-\frac{\gamma^{2}K_{h}}{AGK}-\frac{\gamma^{2}q_{f}}{EI}+\gamma^{2}\beta^{2}Z+\alpha^{2}Z}{\alpha (\gamma^{2}+Z)}+\frac{\alpha q_{f}}{AGK}+\frac{K_{h}}{\alpha (AGK)}\;))+\\ & (-\sin(\alpha L))(\frac{-\frac{\gamma^{2}\alpha^{2}q_{f}}{AGK}\;-\frac{\gamma^{2}K_{h}}{AGK}-\frac{\gamma^{2}q_{f}}{EI}+\gamma^{2}\beta^{2}Z+\alpha^{2}Z}{\gamma^{2}+Z}+\frac{\alpha^{2}q_{f}}{AGK}+\frac{K_{h}}{AGK}))+\\ & A_{2}(\frac{1}{EI}((K_{r_{2}}\sin(\alpha L))(\frac{\frac{\gamma^{2}q_{f}(\alpha^{2}-\beta^{2})}{AGK}+\frac{\gamma^{2}K_{h}}{AGK}+\frac{\gamma^{2}q_{f}}{EI}+\gamma^{2}(-\beta^{2})-\alpha^{2}Z}{\alpha (\gamma^{2}+Z)}-\frac{\alpha q_{f}}{AGK}-\frac{K_{h}}{\alpha (AGK)}))+\\ & \cos(\alpha L)(\frac{\frac{\gamma^{2}\alpha^{2}q_{f}}{AGK}+\;\frac{\gamma^{2}K_{h}}{AGK}+\frac{\gamma^{2}q_{f}}{EI}-\gamma^{2}\beta^{2}Z-\alpha^{2}Z}{\gamma^{2}+Z}-\frac{\alpha^{2}q_{f}}{AGK}-\frac{K_{h}}{AGK}))+\\ & A_{3}\frac{1}{EI}((K_{r_{2}}\cosh(\alpha L))(\frac{-\frac{\gamma^{2}\beta^{2}q_{f}}{AGK}+\frac{\gamma^{2}K_{h}}{AGK}+\frac{\gamma^{2}q_{f}}{EI}+\gamma^{2}\alpha^{2}+\beta^{2}Z}{\beta (\gamma^{2}+Z)}+\frac{\beta q_{f}}{AGK}-\frac{K_{h}}{\beta (AGK)}\;))+\\ & \sinh(\beta L)(\frac{-\frac{\gamma^{2}\beta^{2}q_{f}}{AGK}+\;\frac{\gamma^{2}K_{h}}{AGK}+\frac{\gamma^{2}q_{f}}{EI}+\gamma^{2}\alpha^{2}+\beta^{2}Z}{\gamma^{2}+Z}+\frac{\beta^{2}q_{f}}{AGK}-\frac{K_{h}}{AGK})+\\ & A_{4}(\frac{1}{EI}((K_{r_{2}}\sinh(\beta L))(\frac{-\frac{\gamma^{2}\beta^{2}q_{f}}{AGK}+\frac{\gamma^{2}K_{h}}{AGK}+\frac{\gamma^{2}q_{f}}{EI}+\gamma^{2}\alpha^{2}Z+\beta^{2}Z}{\beta (\gamma^{2}+Z)}+\frac{\beta r}{AGK}-\frac{K_{h}}{\zeta (AGK)}\;))+\\ & \cosh(\beta L)(\frac{-\frac{\gamma^{2}\beta^{2}q_{f}}{AGK}+\;\frac{\gamma^{2}K_{h}}{AGK}+\frac{\gamma^{2}q_{f}}{EI}+\gamma^{2}\alpha^{2}Z+\beta^{2}Z}{\gamma^{2}+Z}+\frac{\beta^{2}q_{f}}{AGK}-\frac{K_{h}}{AGK}))=0 \end{array}$$ (23)

where Z and γ are stated as


$$\begin{array}{c} \gamma =\sqrt{\frac{E}{GK}} \end{array}$$

and


$$\begin{array}{c} Z=\frac{q_{f}}{AGK}+1 \end{array}$$

The results given by Eqs. (20–23) establish a system of 4 equations with 4 variables $A_{1},A_{2},A_{3},$ and *A*_4_, yielding the boundary conditions (2a- 2d). Therefore, a non-trivial solution needs the coefficient matrix determinant to be 0, giving the characteristic solution. It is important to note that the characteristic equation can be utilized to find the eigenvalues β and α. The explicit values of α or β can be determined when α is expressed as a function of β or otherwise. The following process is employed to evaluate the eigenvalues.

The following expressions are extracted by solving Eq. (15) for *E*_1_, *E*_2_, and *E*_3_.


$$\begin{array}{cc} & E_{1}=\frac{\frac{Z(\alpha^{2}-\beta^{2})}{2}+\frac{q_{f}\gamma^{2}}{2EIZ}-\frac{K_{h}\gamma^{2}}{2KGAZ}}{Z+\gamma^{2}}\\ & E_{2}=\frac{E_{1}\gamma^{2}}{Z}+\frac{K_{h}\gamma^{2}}{2KGAZ}+\frac{q_{f}\gamma^{2}}{2EIZ^{2}}\\ & E_{3}=\frac{Z^{2}\alpha^{2}\beta^{2}+4\alpha^{2}\eta^{2}\gamma^{4}+4Z\gamma^{2}\alpha^{4}+4Z\gamma^{2}\beta^{4}}{4(Z+\gamma^{2}{)}^{2}}+\frac{\gamma^{2}}{Z}(\frac{q_{f}\gamma^{2}}{EIZ(Z+\gamma^{2})}{)}^{2}+\frac{2\gamma^{2}}{Z}\frac{Z(\alpha^{2}-\beta^{2})}{Z+\gamma^{2}}\frac{q_{f}\gamma^{2}}{EIZ(Z+\gamma^{2})}\\ & +\frac{\gamma^{2}}{Z(Z+\gamma^{2}{)}^{2}}(\frac{K_{h}\gamma^{2}}{KGAZ}{)}^{2}-\frac{2\gamma^{2}}{Z}\frac{2(\alpha^{2}-\beta^{2})+\frac{q_{f}\gamma^{2}}{EIZ}}{Z+\gamma^{2}}\frac{K_{h}\gamma^{2}}{KGAZ(Z+\gamma^{2})}+(\frac{\gamma^{2}K_{h}}{KGAZ^{2}}+\frac{q_{f}\gamma^{2}}{EIZ^{2}})\\ & \left(\frac{Z(\alpha^{2}-\beta^{2})+\frac{q_{f}\gamma^{2}}{EIZ}-\frac{K_{h}\gamma^{2}}{KGAZ}}{Z+\gamma^{2}}\right) \end{array}$$ (24)

In the following expression for β, we take the ratio of *E*_3_ to *E*_1_ by using Eqs. (15) and (24).


$$\begin{array}{c} \beta =\sqrt{\frac{H-M}{h\sqrt{Z}}} \end{array}$$ (25)

where


$$\begin{array}{cc} M=\frac{1}{2\gamma \sqrt{Z}}( & \;Z(\gamma^{2}+Z)\left(-\frac{q_{f}K_{h}}{A^{2}G^{2}K^{2}Z^{2}}-\frac{EK_{h}}{AG^{2}K^{2}Z^{2}}+\frac{K_{h}}{AGKZ^{2}}+\frac{A}{IZ^{2}}\right)\\ + & \;\frac{2\gamma^{4}K_{h}}{AGKZ}-\frac{\gamma^{2}K_{h}Z(\gamma^{2}+Z)}{AGKZ^{2}}\\ + & \;\frac{2\gamma^{4}q_{f}}{EIZ}-\frac{\gamma^{2}q_{f}Z(\gamma^{2}+Z)}{EIZ}\\ + & \;\gamma^{4}\alpha^{2}+\alpha^{2}Z^{2}) \end{array}$$ (26)

$$\begin{array}{cc} & H^{2}=((-\frac{q_{f}K_{h}}{A^{2}G^{2}K^{2}Z^{2}}-\frac{EK_{h}}{A\;G^{2}K^{2}Z^{2}}+\frac{K_{h}}{AGKZ^{2}}+\frac{s^{2}}{Z^{2}})((\gamma^{2}+Z)(-\frac{\gamma^{2}K_{h}}{AGKZ}-\frac{\gamma^{2}q_{f}}{EIZ}+\alpha^{2}Z)+\\ & \frac{K_{h}(\gamma^{2}+Z{)}^{2}}{AGKZ}+\frac{q_{f}(\gamma^{2}+Z{)}^{2}}{EIZ})-\frac{\gamma^{2}K_{h}(\gamma^{2}\;+Z)(-\frac{\gamma^{2}K_{h}}{AGKZ}-\frac{\gamma^{2}q_{f}}{EIZ}+\alpha^{2}Z)}{AGKZ^{2}}-\frac{1}{EIZ}(\gamma^{2}q_{f}(\gamma^{2}+Z)\\ & (-\frac{\gamma^{2}K_{h}}{AGKZ}-\frac{\gamma^{2}q}{\;}EIZ+\alpha^{2}Z))-\frac{\left(\gamma^{2}+Z{)}^{2}(q_{f}K_{h}\right)}{AEIGKZ^{2}}+\frac{2\gamma^{4}K_{h}(\alpha^{2}Z-\frac{\gamma^{2}q_{f}}{EIZ})}{AGKZ^{2}}-\frac{Z}{\gamma^{2}(\frac{\gamma^{2}K_{h}}{AGK\gamma }{)}^{2}}+\\ & \frac{2\gamma^{2}p^{2}\;(\gamma^{2}q_{f})}{EIZ}-\frac{\gamma^{2}(\frac{\gamma^{2}q_{f}}{EIZ}{)}^{2}}{Z}-\frac{K_{h}(\gamma^{2}+Z{)}^{2}}{EIZ^{2}}-\gamma^{2}\alpha^{4}Z)+M^{2} \end{array}$$

and


$$\begin{array}{c} s=L\sqrt{\frac{A}{I}} \end{array}$$

Here *s* is the slenderness ratio, and the characteristic equation for the Timoshenko beam depends on *s* and γ. Therefore, the eigenvalues depend on both geometrical and physical properties. As stated by the above process, the characteristic equation, along with Eq. (25), gives α and η with the help of the root-finding technique. The eigenfrequencies can be determined by using Eq. (24). It is crucial to note that characteristic equations for Rayleigh and shear models are derived by neglecting the effects of shear deformation and rotary inertia, respectively, from Eqs. (1a-1b). It is also feasible to find the characteristic equation for Euler-Bernoulli beams as a specific case by removing bo). It is alth rotary and shear deformations from the coupled equations. (1a-1b). The equation (8) is a second-order linear differential equation along constants. It is solved by determining the characteristic equation and then supposing a solution of the form $T(t)=e^{r_{c}t}$, in which $r_{c}$ is a constant. When we substitute this into the Eq. (8), we obtain


$$\begin{array}{c} (\rho A+\rho I)r_{c}^{2}e^{r_{c}t}+F_{D}r_{c}e^{r_{c}t}+(\rho A+\rho I)\omega^{2}e^{r_{c}t}=0 \end{array}$$ (27)

We get the characteristic equation by factoring out $e^{r_{c}t}$ as follows:


$$\begin{array}{c} (\rho A+\rho I)r_{c}^{2}+F_{D}r_{c}+(\rho A+\rho I)\omega^{2}=0 \end{array}$$ (28)

To solve for $r_{c}$, use the quadratic formula:


$$\begin{array}{c} r_{c}=\frac{-F_{D}\pm \sqrt{\left(F_{D}{)}^{2}-4((\rho A+\rho I{)}^{2}\omega^{2}\right)}}{2(\rho A+\rho I)} \end{array}$$ (29)

If Eq. (29) is reduced, we get


$$\begin{array}{c} r_{c}=-\xi \omega \pm \omega \sqrt{\xi^{2}-1} \end{array}$$ (30)

or


$$\begin{array}{c} r_{c}=-\xi \omega \pm \omega_{d} \end{array}$$ (31)

And


$$\begin{array}{c} \omega_{d}=\omega \sqrt{\xi^{2}-1} \end{array}$$ (32)

where ξ presents the damping ratio, which can be shown as follows, and $\omega_{d}$ depicts the damped frequency:


$$\begin{array}{c} \xi =\frac{F_{D}}{c_{cm}} \end{array}$$ (33)

and


$$\begin{array}{c} c_{cm}=2(\rho A+\rho I)\omega \end{array}$$ (34)

Remarkable understanding of the system’s response is given by the ξ. Whenever the system is not damped, as is the specific case of ξ=0, the damping term of the differential equation vanishes. The general solution to the Eq. (8) in the damped state is expressed as:


$$\begin{array}{c} T_{1}(t)=e^{-\xi \omega t}(Ee^{\omega_{d}t}\pm Fe^{-\omega_{d}t}) \end{array}$$ (35)

F and E stand for constants, which are obtained by initial conditions. This method makes it simple to conduct a thorough analysis of the beam’s response and structural behavior and provide a thorough grasp of how it functions in various situations.

## Formulation of finite elements method

The finite element method is broadly accepted as one of the most robust and effective techniques to perform structural analysis. The core principle of finite element analysis is the idea that every complicated engineering problem may be approximately solved by dividing a larger complex structure into smaller, non-overlapping, simple-geometry components known as finite elements or elements. These structures are described by complex partial differential equations that may be simplified to a set of linear equations that are simple to solve. In the instance of a Timoshenko beam, the finite element method procedure starts by dividing the beam into finite elements. The weight functions *w*_1_ and *w*_2_ correspond to the displacement *z*(*x*,*t*) and the rotation ψ, respectively. These weight functions are then multiplied to the coupled differential equations (1a−1b). Hence, resulting in the weak forms is required for the finite element formulation.


$$\begin{array}{cc} & \int_{0}^{L}-w_{1}\left(\rho Az_{tt}-KGA(z_{xx}-\psi_{x})+K_{h}z-q_{f}z_{xx}+F_{D}z_{t}\right)dx=\int_{0}^{L}w_{1}\rho Az_{tt}dx-w_{1}KGA\\ & \left(z_{x}-\psi \right){|}_{0}^{L}+\int_{0}^{L}w_{1,x}KGA(z_{x}-\psi )dx+\int_{0}^{L}K_{h}w_{1}zdx-w_{1}q_{f}z_{x}{|}_{0}^{L}+\int_{0}^{L}w_{1,x}q_{f}z_{x}dx+F_{D}\int_{0}^{L}w_{1}z_{t}dx=0 \end{array}$$ (36)

$$\begin{array}{cc} & \int_{0}^{L}-w_{2}\left(EI\psi_{xx}+KGA(z_{x}-\psi )+\rho I\psi_{tt}\right)dx=-w_{2}EI\left(\psi_{x}\right){|}_{0}^{L}+\int_{0}^{L}w_{2,x}EI\psi_{x}dx\\ & +\int_{0}^{L}w_{2}KGA\left(\psi -z_{x}\right)dx-\int_{0}^{L}\rho Iw_{2}\left(\psi_{tt}\right)dx=0 \end{array}$$ (37)

After obtaining the weak form for coupled differential equations, interpolation functions for *z* and ψ are incorporated. It is presumed that *z* and ψ are presented by general quadratic and cubic interpolation shape functions, respectively, as defined in Eqs. (38)–(39).


$$\begin{array}{c} z=\left[1\;\;\;\left(\frac{x}{L}\right)\;\;\;{\left(\frac{x}{L}\right)}^{2}\;\;\;{\left(\frac{x}{L}\right)}^{3}\right]\left[\begin{array}{cccc} 1 & 0 & 0 & 0\\ b_{11} & b_{21} & b_{31} & b_{41}\\ b_{12} & b_{22} & b_{32} & b_{42}\\ b_{13} & b_{23} & b_{33} & b_{43} \end{array}\right]\left[\begin{array}{c} z_{1}\\ \psi_{1}\\ z_{2}\\ \psi_{2} \end{array}\right] \end{array}$$ (38)

and


$$\begin{array}{c} \psi =\left[1\;\;\;\left(\frac{x}{L}\right)\;\;\;{\left(\frac{x}{L}\right)}^{2}\right]\left[\begin{array}{cccc} 0 & 1 & 0 & 0\\ c_{11} & c_{21} & c_{31} & c_{41}\\ c_{12} & c_{22} & c_{32} & c_{42} \end{array}\right]\left[\begin{array}{c} z_{1}\\ \psi_{1}\\ z_{2}\\ \psi_{2} \end{array}\right] \end{array}$$ (39)

The coefficients $c_{ij}$ and $b_{ij}$ are unknown initially. *z*_1_, $\psi_{1}$, *z*_2_, and $\psi_{2}$ represent the rotations and nodal displacements at the ends of the beam. By requiring that (*z*(*x* = *L*)=*z*_2_) and $(\psi (x=L)=\psi_{2}$), four coefficients from each set $c_{ij}$ and $b_{ij}$ can be found in terms of the rest of twelve coefficients. Inserting the shape functions into the Eqs. (36–37) and by solving them, we get the remaining coefficients. The shape functions are represented in Eqs. (40) and (41), respectively:


$$\begin{array}{c} {\left[N_{z}\right]}^{T}=\left[\begin{array}{c} \frac{1}{1+\left\{\phi \right\}}\left\{2\;\frac{x^{3}}{L^{3}}-3\;\frac{x^{2}}{L^{2}}-\frac{\left\{\phi \right\}x}{L}+1+\left\{\phi \right\}\right\}\\ \frac{L}{1+\left\{\phi \right\}}\left\{\frac{x^{3}}{L^{3}}-\frac{x^{2}}{L^{2}}\left(2+\frac{\left\{\phi \right\}}{2}\right)+\frac{x}{L}\left(1+\frac{\left\{\phi \right\}}{2}\right)\right\}\\ -\frac{1}{1+\left\{\phi \right\}}\left\{2\;\frac{x^{3}}{L^{3}}-3\;\frac{x^{2}}{L^{2}}-\frac{\left\{\phi \right\}x}{L}\right\}\\ \frac{L}{1+\left\{\phi \right\}}\left\{\frac{x^{3}}{L^{3}}-\frac{x^{2}}{L^{2}}\left(1-\frac{\left\{\phi \right\}}{2}\right)-\frac{\left\{\phi \right\}x}{2\;L}\right\} \end{array}\right] \end{array}$$ (40)

$$\begin{array}{c} {\left[N_{\psi }\right]}^{T}=\left[\begin{array}{c} \;\frac{6}{\left(1+\left\{\phi \right\}\right)L}\left\{\frac{x^{2}}{L^{2}}-\frac{x}{L}\right\}\\ \frac{1}{1+\left\{\phi \right\}}\left\{\;\frac{x^{2}}{L^{2}}-\frac{\left(4+\left\{\phi \right\}\right)x}{L}+1+\left\{\phi \right\}\right\}\\ -\;\frac{6}{\left(1+\left\{\phi \right\}\right)L}\left\{\frac{x^{2}}{L^{2}}-\frac{x}{L}\right\}\\ \frac{1}{1+\left\{\phi \right\}}\left\{\;\frac{3x^{2}}{L^{2}}-\frac{\left(2-\left\{\phi \right\}\right)x}{L}\right\} \end{array}\right] \end{array}$$ (41)

here ϕ is defined as:


$$\begin{array}{c} \phi =\frac{12EI}{L^{2}KGA} \end{array}$$

In the case of slender and long beams (ϕ=0), the shape function $\left[N_{z}\right]$ simplifies to the cubic Hermitian polynomial, and $\left[N_{\psi }\right]$ corresponds to its derivative. On the other hand, when considering composite or short beams, the shape functions rely on the value of ϕ and are then derived accordingly.

By substituting Eqs. (40–41) into Eqs. (36–37), we get


$$\begin{array}{cc} & \int_{0}^{L}-w_{1}\left(\rho Az_{tt}-KGA(z_{xx}-\psi_{x})+K_{h}-q_{f}z_{xx}+F_{D}z_{t}\right)dx=\int_{0}^{L}\lambda_{i}\rho A\lambda_{j}z_{j,tt}dx-\lambda_{i}KGA(z_{j}\lambda_{j,x}-\\ & \psi_{m}\lambda_{m}){|}_{0}^{L}+\int_{0}^{L}\lambda_{i,x}KGA\left(\nu_{j}\lambda_{j,x}-\psi_{m}\lambda_{m}\right)dx+\int_{0}^{L}K_{h}\lambda_{i}\nu_{j}\lambda_{j}dx-\lambda_{i}q_{f}z_{j}\lambda_{j,x}{|}_{0}^{L}+\\ & \int_{0}^{L}\lambda_{j,x}q_{f}\left(z_{j}\lambda_{j,x}\right)dx+F_{D}\int_{0}^{L}\lambda_{j}\lambda_{i}z_{j,t}dx=0 \end{array}$$ (42)

$$\begin{array}{cc} & \int_{0}^{L}-w_{2}\left(EI\psi_{xx}+KGA(z_{x}-\psi )+\rho I\psi_{tt}\right)dx=-\lambda_{k}EI\lambda_{m,x}\left(\psi_{m}\right){|}_{0}^{L}+\int_{0}^{L}\lambda_{k,x}EI\psi_{m}\lambda_{m,x}dx\\ & +\int_{0}^{L}\lambda_{k}KGA\left(\psi_{m}\lambda_{m}-z_{j}\lambda_{j,x}\right)dx-\int_{0}^{L}\rho I\lambda_{k}\left(\psi_{m,tt}\right)dx=0 \end{array}$$ (43)

Equations (42–43) can be expressed as


$$\begin{array}{c} a[k_{ij}]z_{j}+[m_{ij}]z_{j,tt}+[L_{ij}]z_{j,t}=0 \end{array}$$ (44)

where


$$\begin{array}{c} k_{ij}=\int_{0}^{L}\lambda_{i,x}KGA(\lambda_{j,x}z_{j}-\psi_{m}\lambda_{m})dx+\int_{0}^{L}K_{h}\lambda_{j}\lambda_{i}z_{j}dx+\int_{0}^{L}\lambda_{j,x}q_{f}\lambda_{j,x}z_{j}dx+\\ \int_{0}^{L}\lambda_{k,x}EI\psi_{m}\lambda_{m,x}dx+\int_{0}^{L}\lambda_{k}KGA(\psi_{m}\lambda_{m}-z_{j}\lambda_{j,x})dx \end{array}$$

$$\begin{array}{c} m_{ij}=\int_{0}^{L}z_{j,tt}\lambda_{i}\rho A\lambda_{j}dx-\int_{0}^{L}\rho I\lambda_{k}\left(\psi_{m,tt}\right)dx \end{array}$$

and


$$\begin{array}{c} L_{ij}=F_{D}\int_{0}^{L}\lambda_{j}\lambda_{i}z_{j,t}dx \end{array}$$

Here $m_{ij}$, $k_{ij}$, and $L_{ij}$, denote the mass, stiffness, and damped matrices, respectively.The SSF is employed to the equation (44). The associated state vector 𝐱 is defined as follows:


$$\begin{array}{c} 𝐱=[\begin{array}{c} x_{1}\\ x_{2} \end{array}]=[\begin{array}{c} z\\ z_{t} \end{array}] \end{array}$$ (45)

The displacement *z* and its derivative $z_{t}$ are represented as state variables *x*_1_ and *x*_2_, respectively. By this formulation, the second-order equation is reformulated as a first-order equation, which is helpful for numerical methods.

Eq. (44) is substituted into the equation (45) to get:


$$\begin{array}{c} a[k_{ij}]{𝐱}_{1}+[m_{ij}]{𝐱}_{2t}+[L_{ij}]{𝐱}_{2}=0 \end{array}$$ (46)

Which is rearranged to give:


$$\begin{array}{c} {𝐱}_{2t}=-[m_{ij}{]}^{-1}([k_{ij}]{𝐱}_{1}+[L_{ij}]{𝐱}_{2}) \end{array}$$ (47)

The state-space form equation could be thus express as:


$$\begin{array}{c} {𝐱}_{t}={\left[\begin{array}{c} {𝐱}_{1}\\ {𝐱}_{2} \end{array}\right]}_{t}=[\begin{array}{c} {𝐱}_{2}\\ -[m_{ij}{]}^{-1}([k_{ij}]{𝐱}_{1}+[L_{ij}]{𝐱}_{2}) \end{array}] \end{array}$$ (48)

This reformulation of the equation to state-space form permits easier numerical calculation. The system is then described as


$$\begin{array}{c} {𝐱}_{t}=A𝐱 \end{array}$$ (49)

Where the state matrix *A* is:


$$\begin{array}{c} A=[\begin{array}{cc} 0 & I\\ -[m_{ij}{]}^{-1}[k_{ij}] & -[m_{ij}{]}^{-1}[L_{ij}] \end{array}] \end{array}$$ (50)

The state-space approach simplifies beam dynamics analysis by treating displacement and velocity as state variables. This concept not only improves system clarity, but also allows for the adoption of computationally efficient numerical integration methodologies, such as the fourth-order Runge-Kutta algorithm. The vibrational response can be accurately represented by breaking down the system into two components: displacement (*x*_1_) and velocity (*x*_2_). Furthermore, the SSF allows for the smooth incorporation of damping effects and various complex beam responses, which are typically difficult to manage with other available approaches.

To calculate the damped and undamped natural frequencies of an elastically supported beam, a MATLAB-based code employing the Finite Element Method is created. This makes it easier to generate global stiffness matrices, especially when there are a lot of elements. The eigenvalues are calculated using Eq.(50), which contains the mass, stiffness, and damping matrices. This eigenvalues gives vital information about the system’s dynamic characteristics. Notably, the presence of complex eigenvalues indicates oscillatory behavior. Furthermore, for long beams with Φ=0, the stiffness matrix decreases to that of the Euler-Bernoulli beam, confirming the absence of shear locking. Setting Φ=0 converts all relevant matrices to their EBB equivalents, as the coefficients $m_{ij}$ and $k_{ij}$ are dependent on Φ. Moreover, the Runge–Kutta 4th order method will be employed to simulate and visualize the vibrational behavior of the beam in both damped and undamped conditions.

## Results and discussions

This study used analytical and numerical calculations to investigate the vibrational behaviors of elastically constrained shear, Timoshenko, Euler-Bernoulli, and Rayleigh beams supported by three-parametric elastic foundation models. The study investigates three different damping conditions: undamped, underdamped, and overdamped, to demonstrate how changes in foundation stiffness and energy dissipation affect the dynamic behavior of different beams. The undamped condition is first examined to establish a reference point for the beams’ mode shapes and natural frequencies with no energy loss. These frequencies serve as a baseline for evaluating the effects of foundation stiffness and damping. The underdamped regime is then investigated, with the inclusion of intermediate damping incorporating energy dissipation while the system is oscillating. Finally, the overdamped case shows how considerable damping entirely eliminates oscillations, leaving non-oscillatory behavior regulated by exponential decay. Reference [25] identifies uniform beam qualities for the investigation. The cross-sectional area *A* = 0.0075 m^2^, second moment of inertia $I=14.063\times 10^{-6}\;{\text{m}}^{4}$, length *L* = 1 m, Young’s modulus *E* = 207 GPa, shear correction factor *K* = 0.53066, density $\rho =76,500\;{\text{kg/m}}^{3}$, and shear modulus *G* = 79.5 GPa. The numerical correctness of the finite element method was tested by comparing results to theoretical values.

Tables 1 and 2 show the evaluated natural frequencies for the Shear, Timoshenko, Euler Bernoulli, and Rayleigh Beam resting over a viscoelastic Hetényi foundation for four vibrational modes and various damping values by considering $K_{r_{1}}$, $K_{r_{2}}=0$, $K_{l_{1}}$, $K_{l_{2}}=10^{13}$, $K_{h}=10^{6}$, and $q_{f}=EI$. The foundation stiffness varies from $1\times 10^{4}\;{\text{N/m}}^{2}$ (Table 1) to $1\times 10^{6}\;{\text{N/m}}^{2}$ (Table 2). The imaginary parts of the eigenvalues get larger as the foundation stiffness increases when there is no damping ($F_{D}=0$), which means there is more resistance to bending because of better support. For instance, the first TB-AM mode increases from 113.558ι to 113.802ι, and the Euler Bernoulli beam first mode shifts from 119.309ι to 119.488ι as the foundation stiffness is increased. The impact of this change becomes greater in higher-order modes where the interaction between shear deformation and flexural rigidity is more significant.

**Table 1:** Damped and undamped frequencies of beam models over a Hetenyi Foundation, considering $K_{r_{1}}=K_{r_{2}}=0$, $K_{l_{1}}=K_{l_{2}}=10^{13}$, $K_{h}=10^{4}$ and $q_{f}=EI$.

BM	$\omega_{1}$	$\omega_{2}$	$\omega_{3}$	$\omega_{4}$
$F_{D}=0$				
TB-AM	0±113.558ι	0±398.029ι	0±789.626ι	0±1236.480ι
TB-FEM	0±114.753ι	0±389.782ι	0±795.233ι	0±1246.874ι
SB-FEM	0±115.698ι	0±410.536ι	0±824.399ι	0±1299.378ι
RB-FEM	0±118.216ι	0±439.159ι	0±939.328ι	0±1579.037ι
EBB-FEM	0±119.309ι	0±455.122ι	0±1014.528ι	0±1797.608ι
$F_{D}=2\times 10^{5}$				
TB-AM	−27.687±110.131ι	−27.687±397.065ι	−27.687±789.140ι	−27.687±1236.170ι
TB-FEM	−27.687±111.461ι	−27.687±399.900ι	−27.687±794.820ι	−27.687±1246.622ι
SB-FEM	−27.738±112.323ι	−27.735±409.597ι	−27.736±823.932ι	−27.735±1299.081ι
RB-FEM	−27.739±115.036ι	−27.739±438.399ι	−27.739±939.027ι	−27.739±1578.891ι
EBB-FEM	−27.739±116.035ι	−27.739±454.276ι	−27.739±1014.148ι	−27.739±1797.394ι
$F_{D}=10^{6}$				
TB-AM	−217.618,−59.256	−138.438±373.178ι	−138.438±777.396ι	−138.438±1228.710ι
TB-FEM	−210.266,−62.632	−138.430±378.412ι	−138.430±784.851ι	−138.430±1240.550ι
SB-FEM	−215.181,−62.207	−138.689±386.399ι	−138.682±812.650ι	−138.676±1291.956ι
RB-FEM	−203.772,−68.581	−138.697±419.743ι	−138.697±931.773ι	−138.697±1575.406ι
EBB-FEM	−209.430,−67.963	−138.697±433.473ι	−138.697±1005.003ι	−138.697±1792.249ι
$F_{D}=10^{9}$				
TB-AM	−276875.00, −0.047	−27685.00, −0.572	−276873.00, −2.251	−276870.00, −5.522
TB-FEM	−276541.62,−0.0482	−276460.14, −0.607	−276373.16, −2.450	−276280.59, −6.087
SB-FEM	−276852.93, −0.0482	−276792.79, −0.061	−276729.77, −2.450	−276663.74, −6.087
RB-FEM	−275332.865, −0.051	−276588.707, −0.0746	−272649.616, −3.710	−231858.485, −11.649
EBB-FEM	−277394.185, −0.051	−277393.48, −0.746	−277390.52, −3.710	−277385.599, −11.649

Note: TB-AM = Timoshenko Beam–Analytical Method; TB-FEM = Timoshenko Beam–Finite Element Method; SB-FEM = Shear Beam–Finite Element Method; RB-FEM = Rayleigh Beam–Finite Element Method; EBB-FEM = Euler–Bernoulli Beam–Finite Element Method.

**Table 2:** Damped and undamped frequencies of beam models over a Hetenyi Foundation, considering $K_{r_{1}}=K_{r_{2}}=0$, $K_{l_{1}}=K_{l_{2}}=10^{13}$, $K_{h}=10^{6}$ and $q_{f}=EI$.

BM	$\omega_{1}$	$\omega_{2}$	$\omega_{3}$	$\omega_{4}$
$F_{D}=0$				
TB-AM	0±113.802ι	0±398.740ι	0±791.946ι	0±1241.480ι
TB-FEM	0±114.939ι	0±400.823ι	0±795.258ι	0±1246.889ι
SB-FEM	0±115.888ι	0±410.588ι	0±824.425ι	0±1299.394ι
RB-FEM	0±118.397ι	0±439.205ι	0±939.348ι	0±1579.047ι
EBB-FEM	0±119.488ι	0±455.170ι	0±1014.549ι	0±1797.620ι
$F_{D}=2\times 10^{5}$				
TB-AM	−27.687±110.383ι	−27.687±397.778ι	−27.687±791.462ι	−27.687±1241.170ι
TB-FEM	−27.688±111.654ι	−27.688±399.953ι	−27.688±794.846ι	−27.688±1246.637ι
SB-FEM	−27.737±112.517ι	−27.737±409.651ι	−27.736±823.958ι	−27.734±1299.089ι
RB-FEM	−27.738±115.222ι	−27.738±438.446ι	−27.738±939.047ι	−27.738±1578.903ι
EBB-FEM	−27.739±116.223ι	−27.739±454.324ι	−27.739±1014.170ι	−27.739±1797.406ι
$F_{D}=10^{6}$				
TB-AM	−217.266, −59.609	−138.438±373.937ι	−138.438±779.752ι	−138.438±1233.740ι
TB-FEM	−209.974, −62.924	−138.557±373.889ι	−138.557±777.310ι	−138.547±1240.566ι
SB-FEM	−209.895, −62.494	−138.688±386.456ι	−138.682±812.677ι	−138.676±1291.973ι
RB-FEM	−209.454, −68.899	−138.697±419.791ι	−138.697±931.793ι	−138.697±1575.417ι
EBB-FEM	−209.120, −68.273	−138.697±433.524ι	−138.697±1005.024ι	−138.697±1792.262ι
$F_{D}=10^{9}$				
TB-AM	−276875.00, −0.046	−276875.00, −0.574	−276873.00, −2.265	−276870.00, −5.566
TB-FEM	−277365.62,−0.0484	−277354.9, −0.608	−277349.7, −2.450	−277328.24, −6.087
SB-FEM	−277875.83, −0.0484	−277757.89, −0.608	−277662.9, −2.450	277584.3, −6.087
RB-FEM	−275332.8, −0.051	−277588.7, −0.0746	−272649.6, −3.711	−271858.4, −11.649
EBB-FEM	−277394.1, −0.051	−277393.4, −0.747	−277392.5, −3.711	−277380.5, −11.649

Note: TB-AM = Timoshenko Beam–Analytical Method; TB-FEM = Timoshenko Beam–Finite Element Method; SB-FEM = Shear Beam–Finite Element Method; RB-FEM = Rayleigh Beam–Finite Element Method; EBB-FEM = Euler–Bernoulli Beam–Finite Element Method.

Eigenvalues in the underdamped conditions ($F_{D}=2\times 10^{5}\text{ Ns/m}$ and $F_{D}=10^{6}\text{ Ns/m}$) have a dominant imaginary component while gaining a negative real part. The system is oscillating while dissipating energy progressively. For instance, for $F_{D}=2\times 10^{5}\text{ Ns/m}$, the TB-AM mode shifts to −27.687±110.131ι (Table 1) and −27.687±110.383ι (Table 2). As the damping increases to $F_{D}=10^{6}\text{ Ns/m}$, the real part’s magnitude increases significantly to −217.618 for TB-AM, while the imaginary part contracts, demonstrating stronger damping effects and reduced vibration amplitude. In the underdamped range, the higher the damping, the faster the oscillatory motion decays and the shorter the vibration duration. Eigenvalues that become real numbers characterize the overdamped regime ($F_{D}=10^{9}\text{ Ns/m}$), demonstrating the supremacy of non-oscillatory exponential decay and the total suppression of oscillations. For example, the TB-AM mode under overdamped conditions shows eigenvalues of −276,875.00 and −0.047, signifying that any initial disturbance is damped out almost instantaneously due to the extremely high dissipative effect.

With the parameters $K_{l_{1}}=K_{l_{2}}=10^{12}$, $K_{r_{1}}=K_{r_{2}}=10^{4}$, $q_{f}=EI$, and $K_{h}=10^{5}$, the undamped and damped dynamic behaviors of a Timoshenko beam backed by a viscoelastic Hetényi foundation are illustrated in Figs 3-6. Several values of the damping coefficient are examined, including 0, $2\times 10^{6}$, $4\times 10^{6}$, and 10^8^. For $F_{D}=0$, the beam demonstrates continuous harmonic oscillations, which is an attribute of an ideal undamped system where no energy dissipation takes place, allowing continuous vibration at the system’s natural frequency. When the damping coefficient is increased to $F_{D}=2\times 10^{6}$, the system enters an underdamped regime. In this case, oscillatory motion continues; however, the amplitude gradually decays with time due to partial energy dissipation per vibration cycle. At $F_{D}=4\times 10^{6}$, the system remains underdamped but with a higher damping ratio, leading to a quicker decay of oscillations. Compared to the lower damping case, the vibration response decays more rapidly due to the increased rate of energy dissipation. With $F_{D}=10^{8}$, the system shifts to an overdamped regime. In this situation, oscillatory behavior is completely suppressed, and the beam reverts monotonically to its equilibrium position. The dominant damping force dissipates vibratory energy sufficiently to prevent any oscillations, resulting in a critically stable response. As the damping coefficient increases from 0 to 10^8^, the system response shifts from undamped free vibration to lightly damped underdamped behavior with decreasing amplitude, then to heavily damped underdamped motion with faster decay, and finally to an overdamped response with no oscillations. This development clearly demonstrates the effect of increased damping on the vibratory characteristics of the beam-foundation system. Figs 7-10 and 11-14 show similar trends for different damping coefficient values, indicating consistent dynamic behavior across diverse configurations.

**Fig 3:**
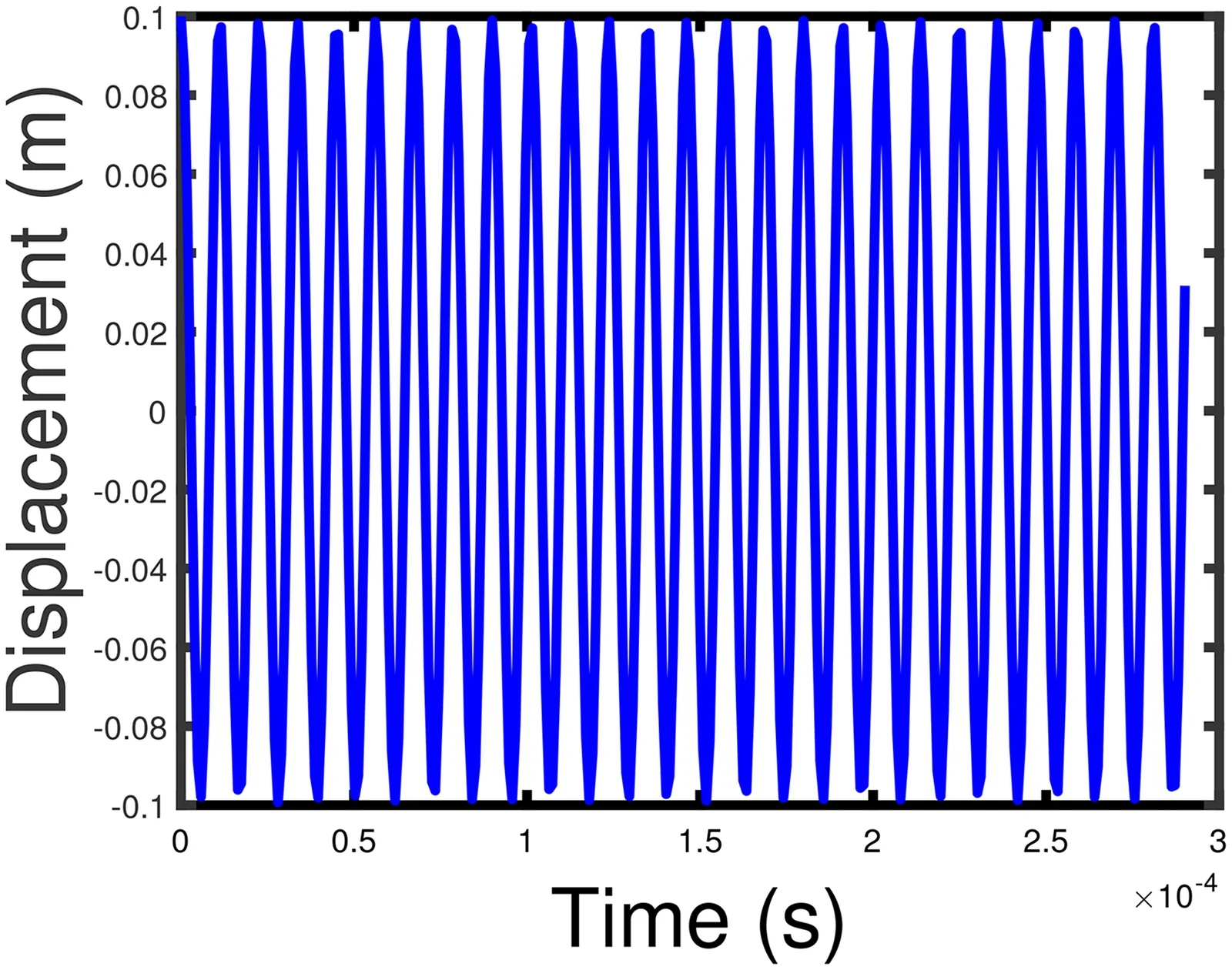
Oscillatory response of the TB over the Hetenyi Foundation by Given that $K_{l_{1}}=K_{l_{2}}=10^{12}$, $K_{r_{1}}=K_{r_{2}}=10^{4}$, $K_{h}=10^{5}$ and adapting $F_{D}=0$.

**Fig 4:**
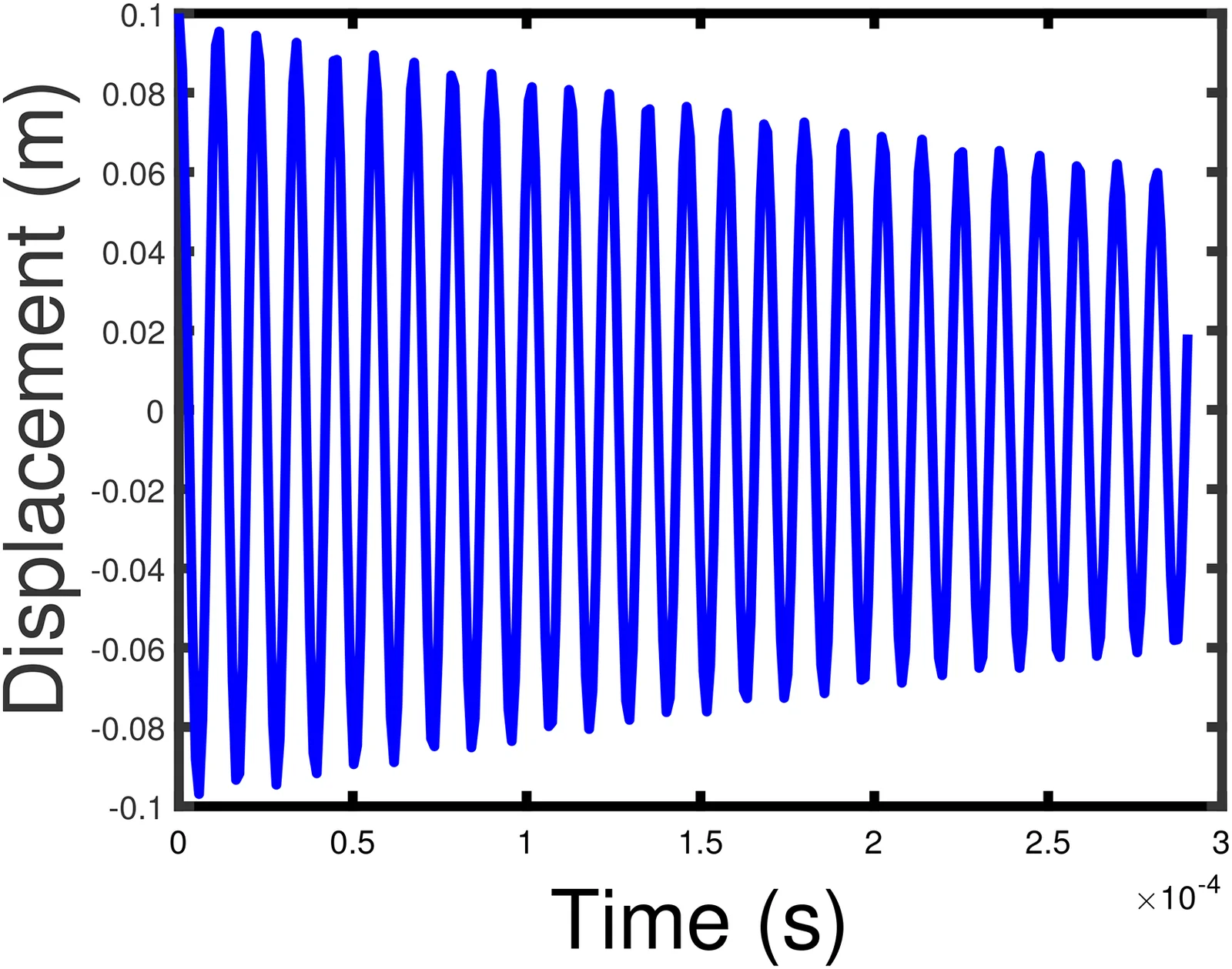
Oscillatory response of the TB over the Hetenyi Foundation by Given that $K_{l_{1}}=K_{l_{2}}=10^{12}$, $K_{r_{1}}=K_{r_{2}}=10^{4}$, $K_{h}=10^{5}$ and adapting $F_{D}=2\times 10^{6}$.

**Fig 5:**
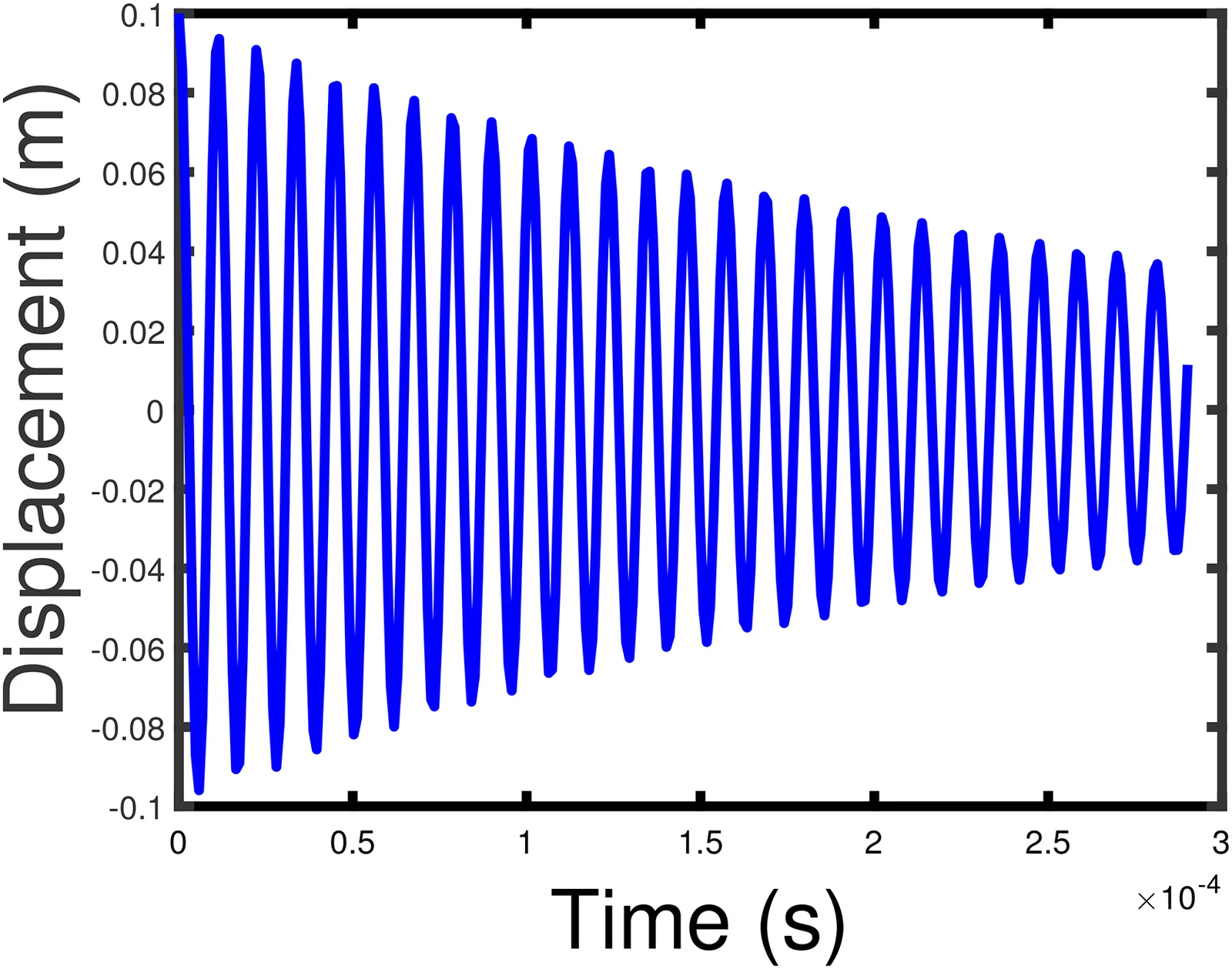
Oscillatory response of the TB over the Hetenyi Foundation by Given that $K_{l_{1}}=K_{l_{2}}=10^{12}$, $K_{r_{1}}=K_{r_{2}}=10^{4}$, $K_{h}=10^{5}$ and adapting $F_{D}=4\times 10^{6}$.

**Fig 6:**
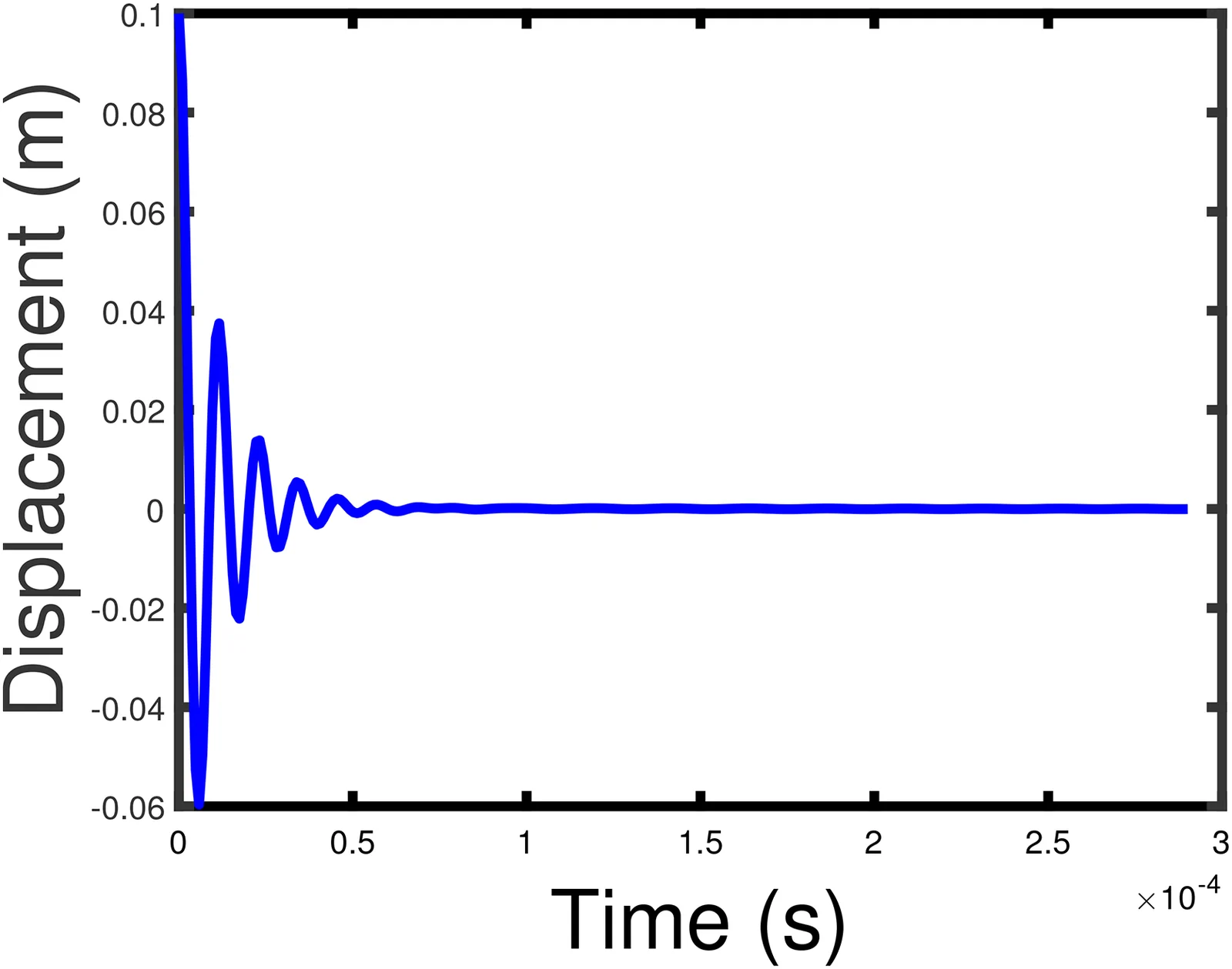
Oscillatory response of the TB over the Hetenyi Foundation by Given that $K_{l_{1}}=K_{l_{2}}=10^{12}$, $K_{r_{1}}=K_{r_{2}}=10^{4}$, $K_{h}=10^{5}$ and adapting $F_{D}=1\times 10^{8}$.

**Fig 7:**
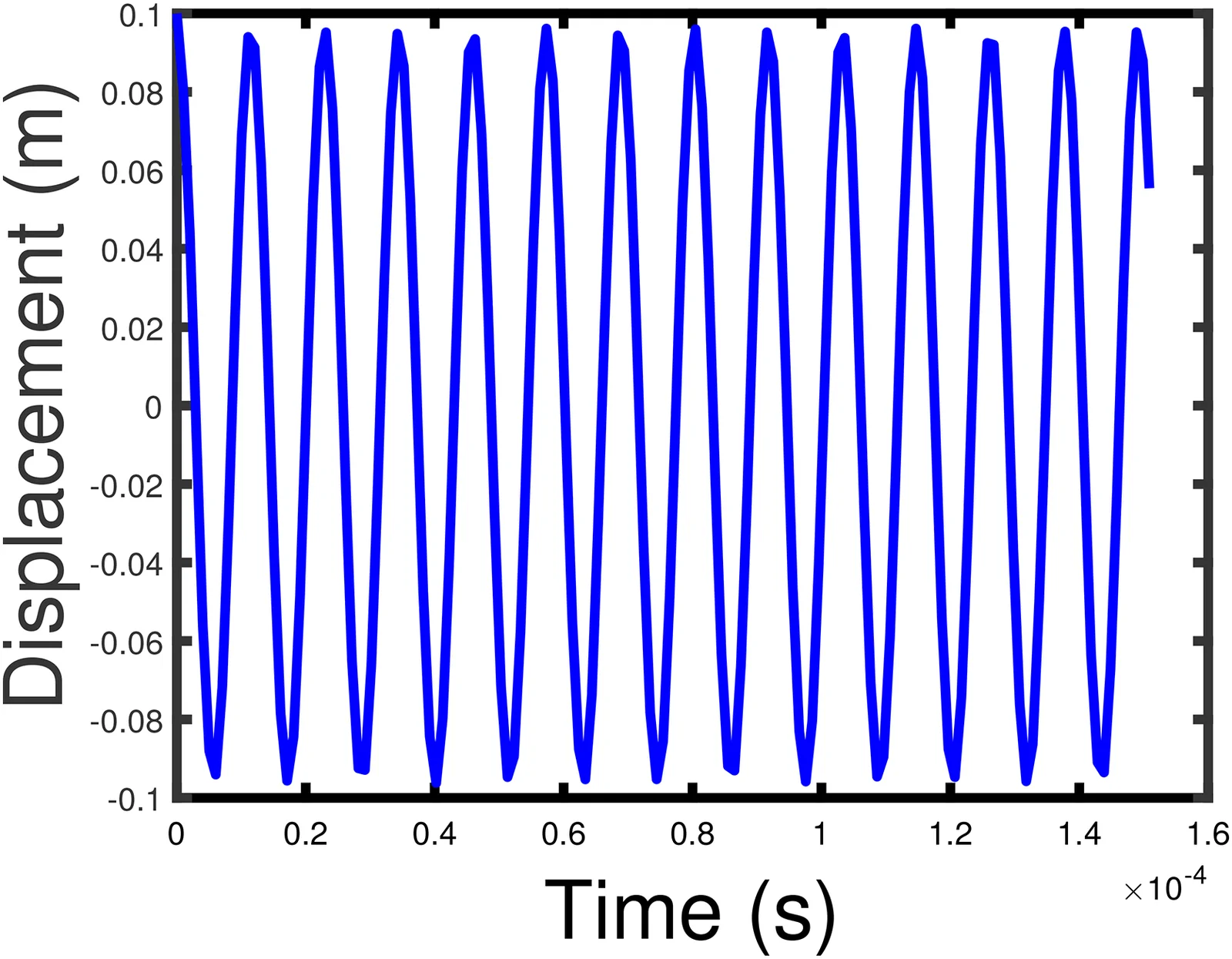
Oscillatory response of the SB over the Hetényi Foundation for given $K_{l_{1}}=K_{l_{2}}=10^{12}$, $K_{r_{1}}=K_{r_{2}}=10^{4}$, and $K_{h}=10^{5}$, with different values of $F_{D}=0$.

**Fig 8:**
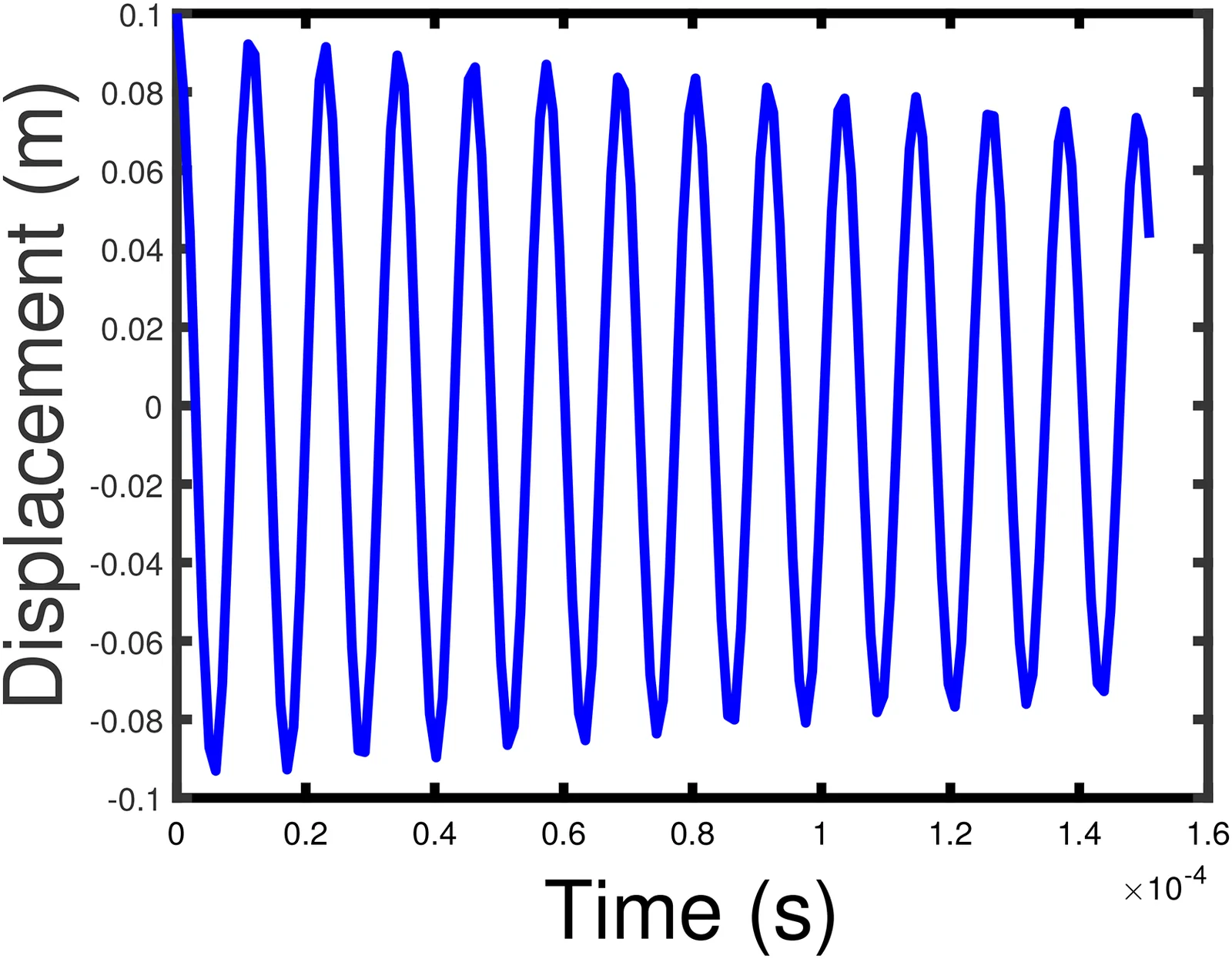
Oscillatory response of the SB over the Hetényi Foundation for given $K_{l_{1}}=K_{l_{2}}=10^{12}$, $K_{r_{1}}=K_{r_{2}}=10^{4}$, and $K_{h}=10^{5}$, with different values of $F_{D}=F_{D}=2\times 10^{6}$.

**Fig 9:**
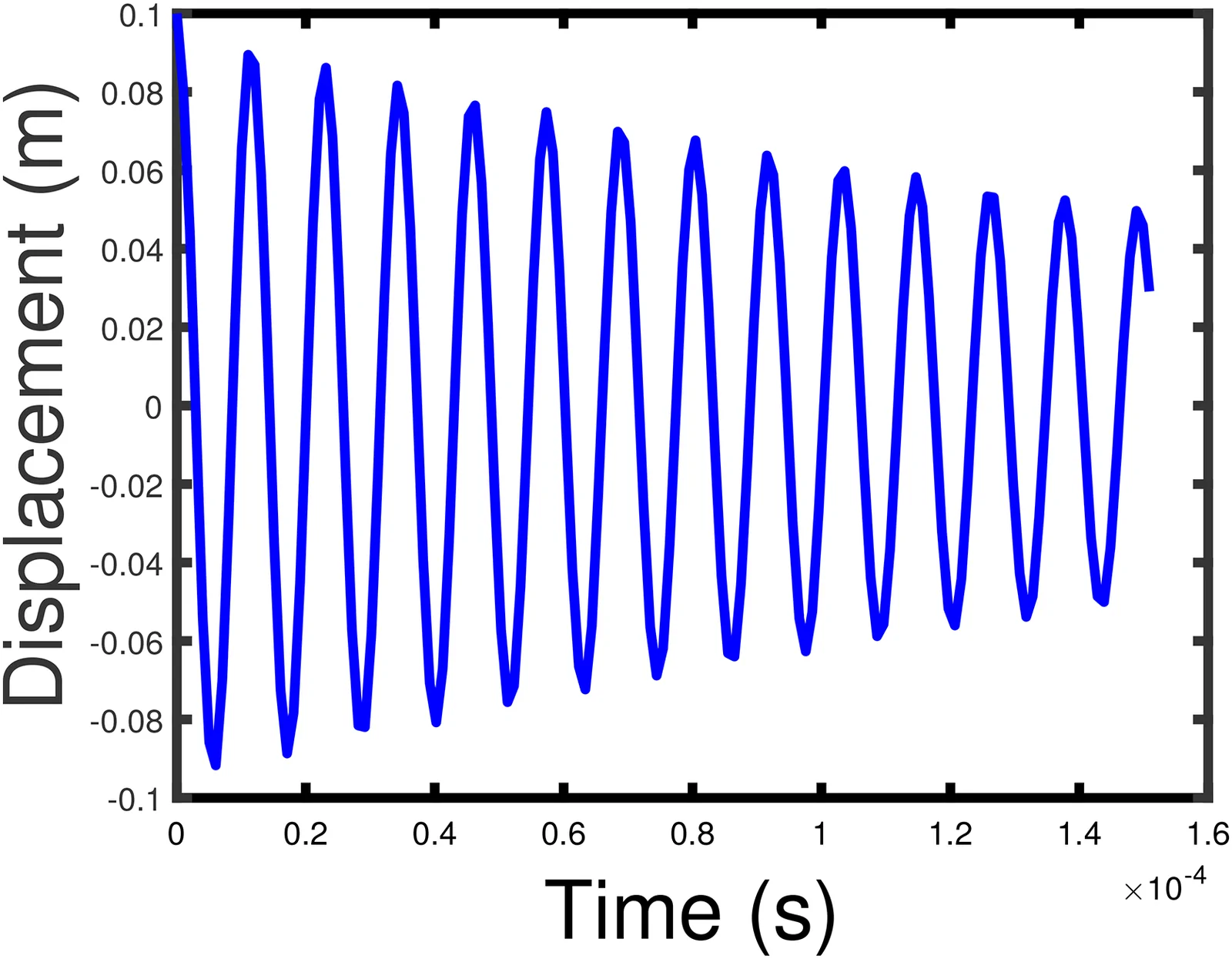
Oscillatory response of the SB over the Hetényi Foundation for given $K_{l_{1}}=K_{l_{2}}=10^{12}$, $K_{r_{1}}=K_{r_{2}}=10^{4}$, and $K_{h}=10^{5}$, with different values of $F_{D}=5\times 10^{6}$.

**Fig 10:**
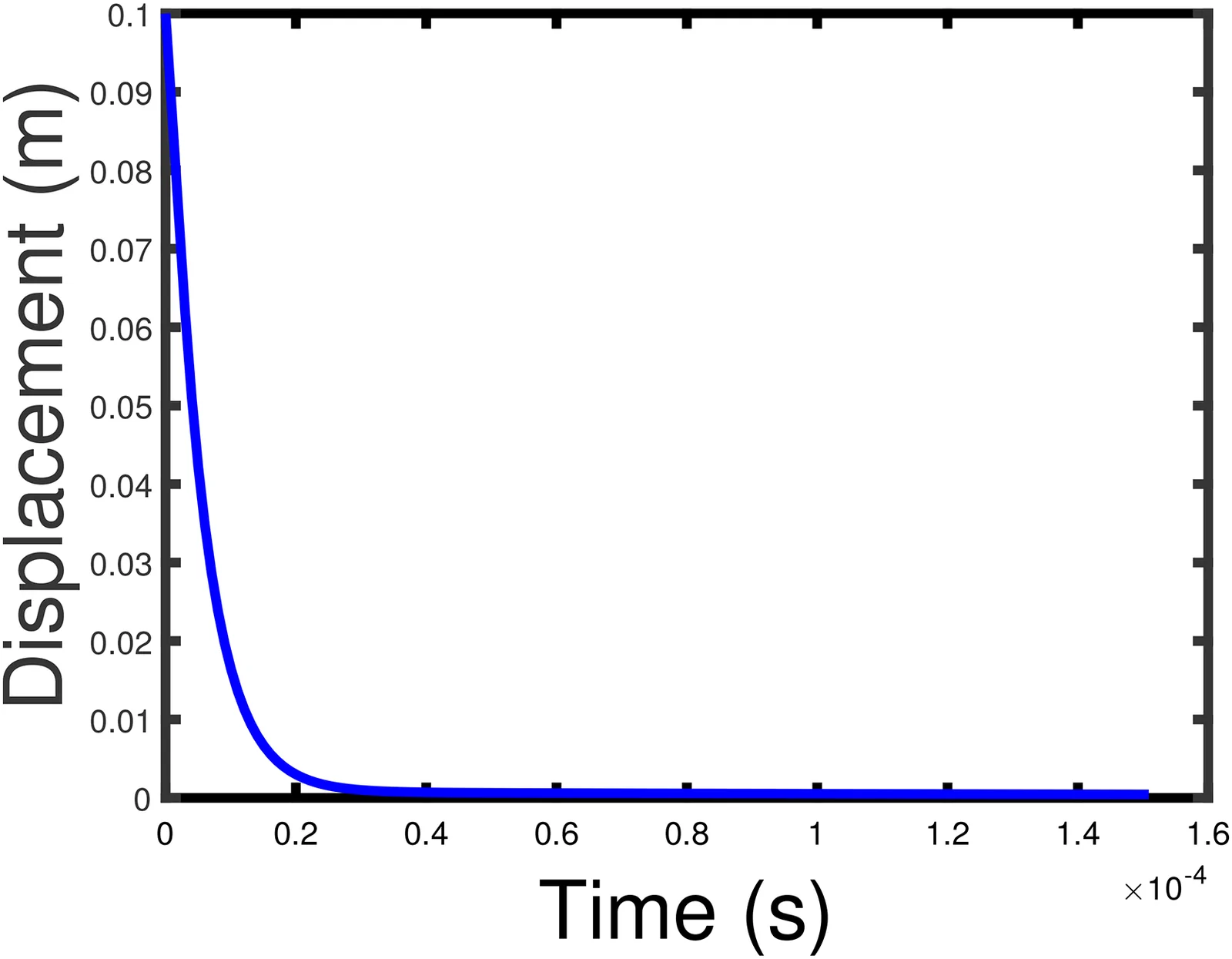
Oscillatory response of the SB over the Hetényi Foundation for given $K_{l_{1}}=K_{l_{2}}=10^{12}$, $K_{r_{1}}=K_{r_{2}}=10^{4}$, and $K_{h}=10^{5}$, with different values of $F_{D}=1\times 10^{9}$.

**Fig 11:**
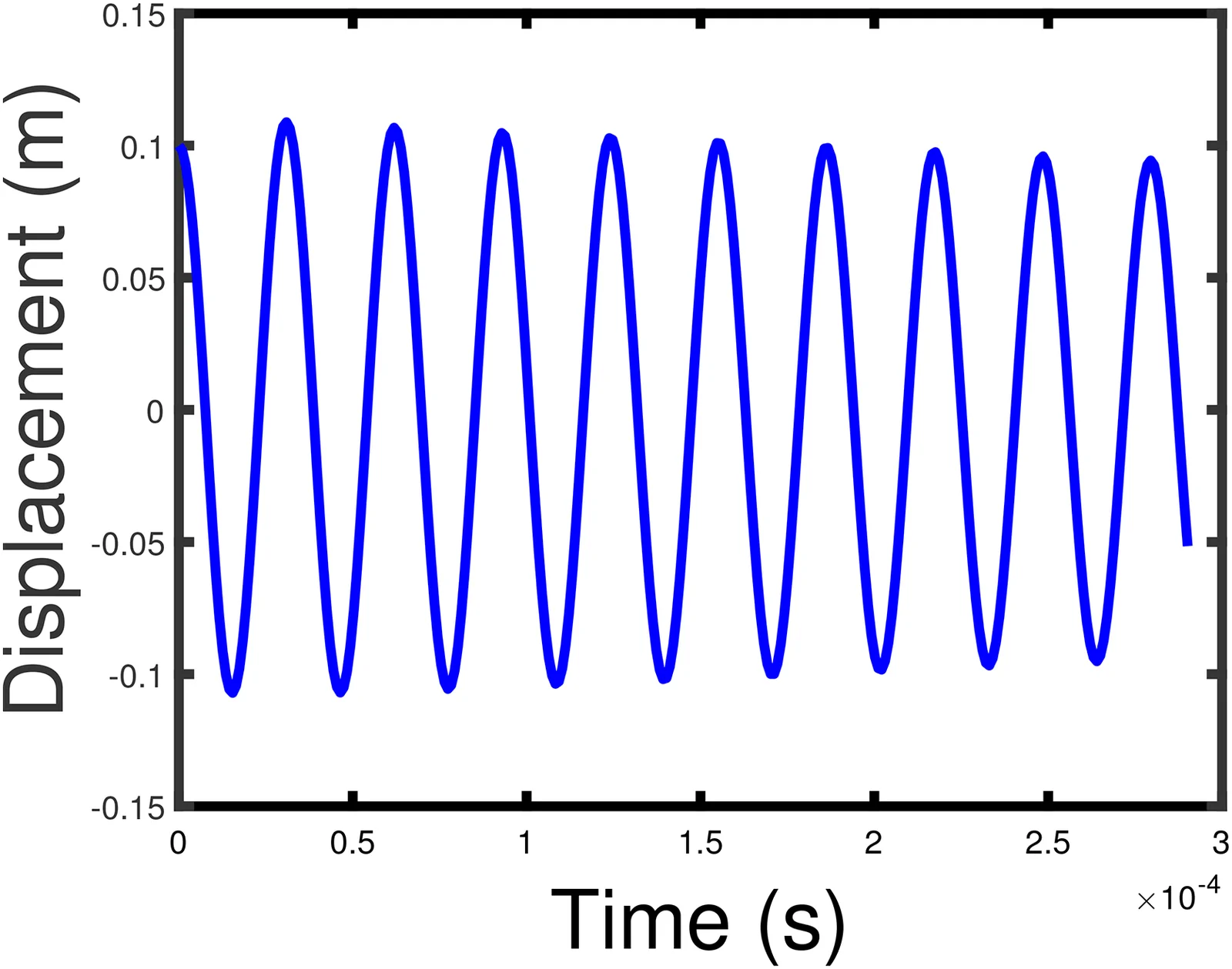
Oscillatory response of the RB over the Hetényi Foundation for given $K_{l_{1}}=K_{l_{2}}=10^{12}$, $K_{r_{1}}=K_{r_{2}}=10^{4}$, and $K_{h}=10^{5}$, with different values of $F_{D}=0$.

**Fig 12:**
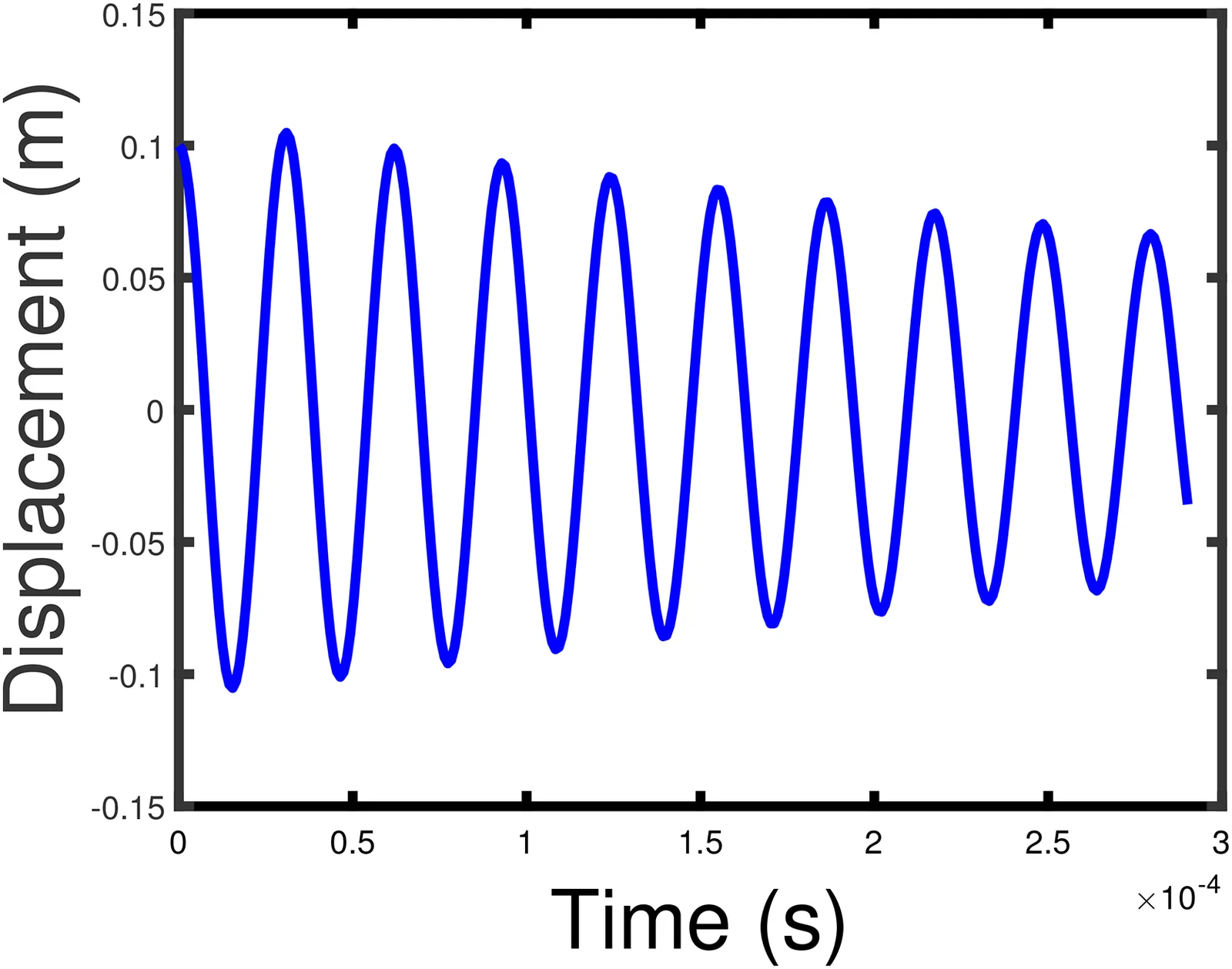
Oscillatory response of the RB over the Hetényi Foundation for given $K_{l_{1}}=K_{l_{2}}=10^{12}$, $K_{r_{1}}=K_{r_{2}}=10^{4}$, and $K_{h}=10^{5}$, with different values of $F_{D}=2\times 10^{7}$.

**Fig 13:**
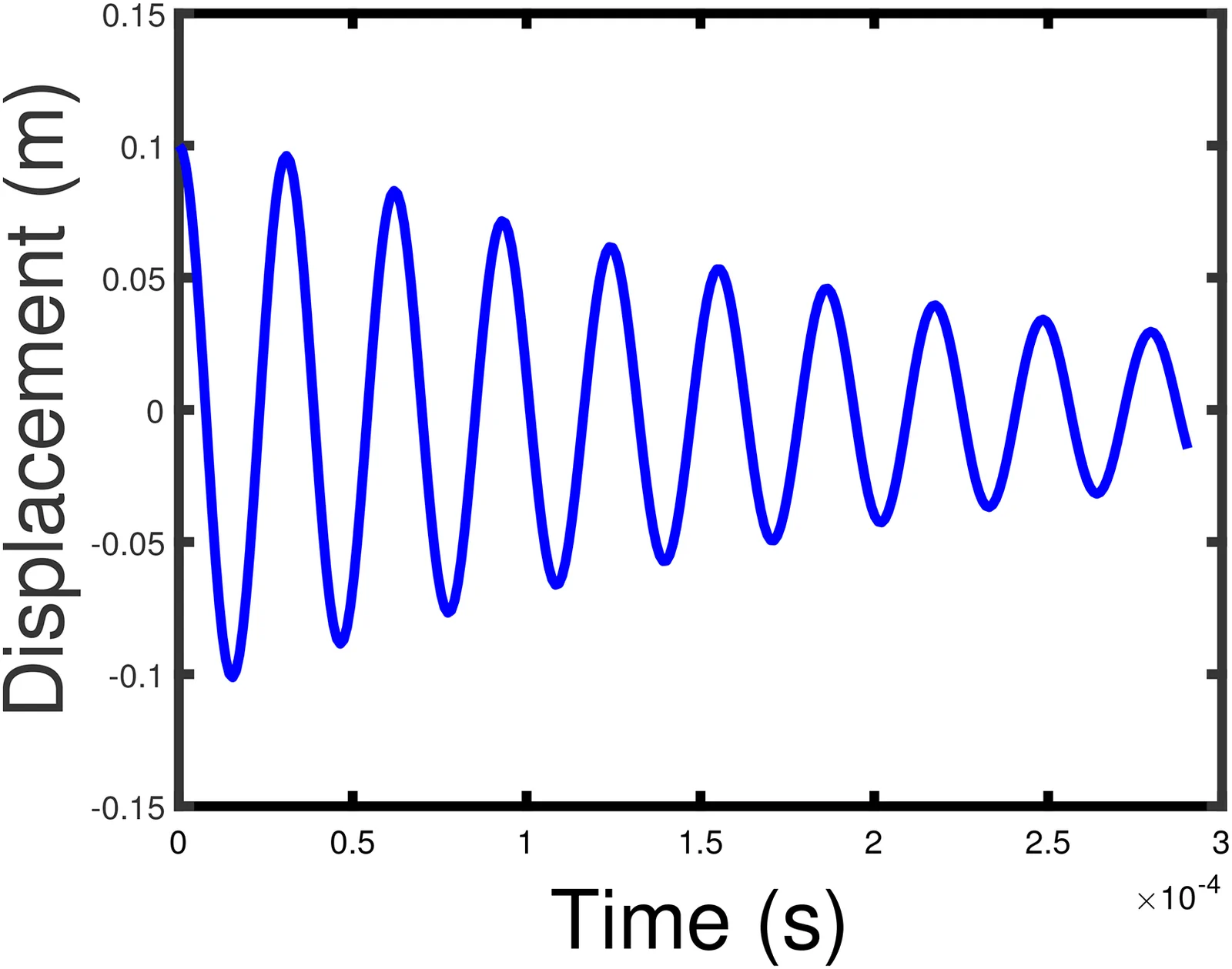
Oscillatory response of the RB over the Hetényi Foundation for given $K_{l_{1}}=K_{l_{2}}=10^{12}$, $K_{r_{1}}=K_{r_{2}}=10^{4}$, and $K_{h}=10^{5}$, with different values of $F_{D}=4\times 10^{7}$.

**Fig 14:**
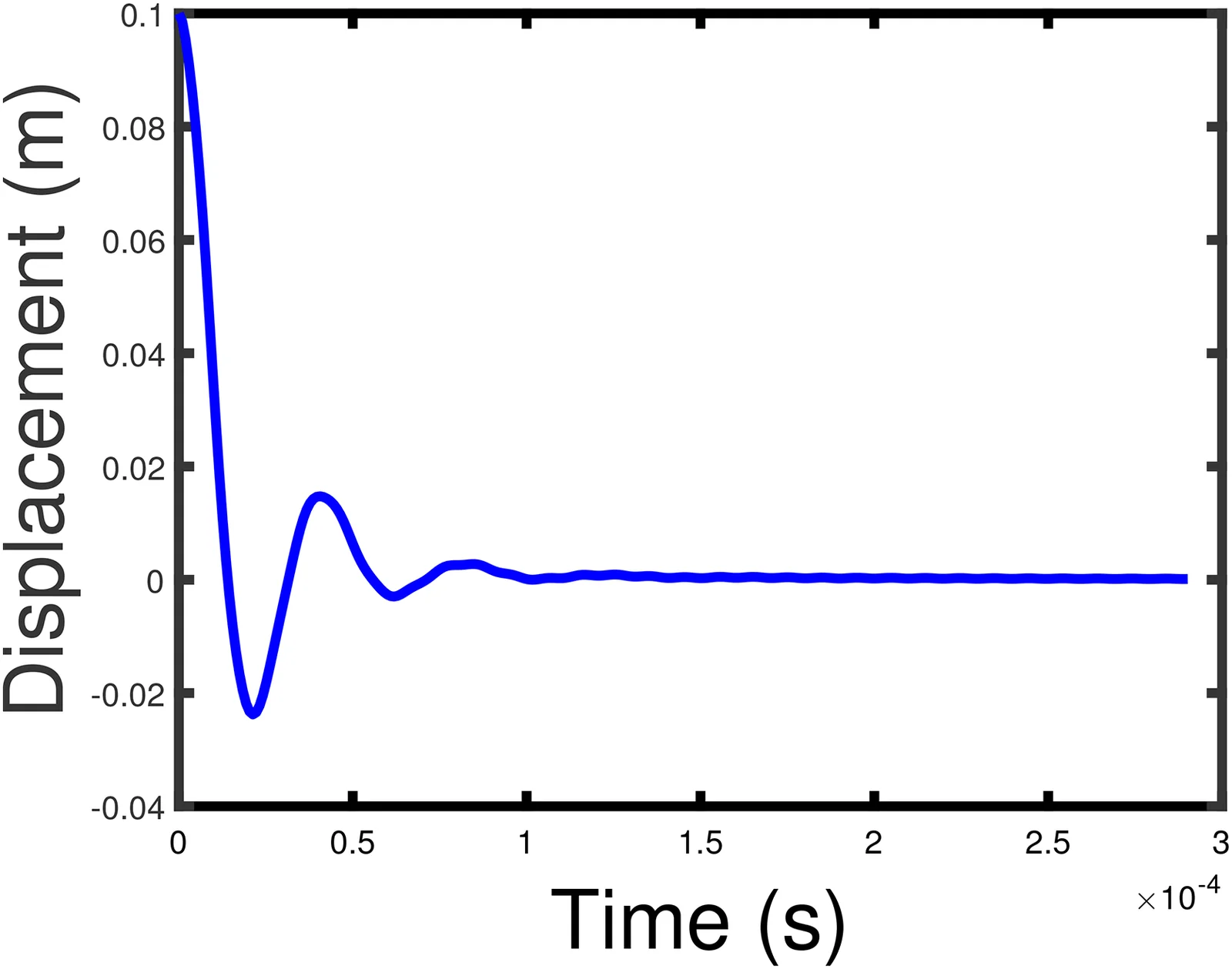
Oscillatory response of the RB over the Hetényi Foundation for given $K_{l_{1}}=K_{l_{2}}=10^{12}$, $K_{r_{1}}=K_{r_{2}}=10^{4}$, and $K_{h}=10^{5}$, with different values of $F_{D}=1\times 10^{10}$.

The dynamic response characteristics of four beam models, Shear, Timoshenko, Euler Bernoulli, and Rayleigh Beam, supported by a Viscoelastic Filonenko-Borodich Foundation, were analyzed by varying damping values and foundation stiffness parameters ($K_{r_{1}}=K_{r_{2}}=K_{l_{1}}=K_{l_{2}}=10^{7}$; $K_{h},q_{f}=10^{4}$ or 10^6^), shown in Tables 3 and 4. In both Tables 3 and 4, the foundation parameters $K_{h}$ and $q_{f}$ increase from $1\times 10^{4}$ to $1\times 10^{6}$ (in their respective units), with a slight but consistent increase in frequencies. Both parameters are varied simultaneously, resulting in a reported shift in frequency caused by their combined effect, rather than due to either parameter alone. For instance, the fundamental TB-AM mode increases from 29.296ι to 32.009ι, while the fourth mode shifts from 661.157ι to 662.752ι. These differences confirm that although the Viscoelastic Filonenko Foundation improves resistance to deflection with increased stiffness. For the undamped case ($F_{D}=0$), all models exhibited purely imaginary eigenvalues (±ωι), representing ideal harmonic oscillation free from any amplitude decay. For the Timoshenko Beam, our numerical implementation was validated by the high agreement between the analytical (TB-AM) and finite element (TB-FEM) solutions. As damping was incorporated ($F_{D}=2\times 10^{5}$ to $4\times 10^{5}$), the system converted to underdamped behavior (ξ<1), defined by complex conjugate eigenvalues (−Re±ωι) demonstrating oscillatory motion with exponential amplitude decline. For example, the TB-AM mode shows eigenvalues of −27.687±9.572ι (Table 3) and −27.688±16.062ι (Table 4). When the damping is doubled to $F_{D}=4\times 10^{5}\text{ Ns/m}$, the real part’s magnitude increases while the imaginary part decreases, indicating faster amplitude decline (e.g., TB-AM second mode: −55.375±67.883ι versus −55.375±70.638ι). The Timoshenko formulation demonstrated more effective energy dissipation compared to the other beam models. At extreme damping ($F_{D}=2\times 10^{8}$), all modes became overdamped (ξ>1), with real, distinct eigenvalues representing pure exponential decay. Figs 15-18 depicts the gradual effect of increasing damping on the dynamic response of a Timoshenko beam placed on a viscoelastic Filonenko-Borodich foundation by considering $K_{l_{1}}=K_{l_{2}}=10^{13}$, $K_{r_{1}}=K_{r_{2}}=10^{13}$, $q_{f}=EI$, and $K_{h}=10^{5}$. Without damping ($F_{D}=0$), the beam demonstrates continuous free oscillations at its natural frequency with no energy loss. Introducing a moderate damping level ($F_{D}=4\times 10^{7}$) induces slight energy dissipation, which reduces the amplitude of the oscillations over time while preserving the underdamped nature of the response. Further increasing the damping coefficient to $F_{D}=8\times 10^{7}$ amplifies the energy dissipation effect, accelerating the decay of oscillatory motion and shifting the system closer to critical damping. When the damping reaches $F_{D}=10^{9}$, the system moves into an overdamped regime, allowing the beam to return monotonically to its static equilibrium position. As the damping magnitude increases, the dynamic behavior transitions from undamped to underdamped and ultimately to overdamped. Figs 19-22 and 23-26 illustrate a similar trend for various values of the damping coefficient, with parameters $K_{l_{l}}=K_{l_{2}}=10^{13}$, $K_{r_{l}}=K_{r_{2}}=0$, and $q_{f}=K_{h}=10^{5}$, respectively, confirming the consistent behavior across different configurations.

**Table 3:** Damped and undamped frequencies of beam models over a Filonenko-Borodich Foundation, considering $K_{r_{1}}=K_{r_{2}}=K_{l_{1}}=K_{l_{2}}=10^{7}$ and $K_{h}=q_{f}=10^{4}$.

BM	$\omega_{1}$	$\omega_{2}$	$\omega_{3}$	$\omega_{4}$
$F_{D}=0$				
TB-AM	0±29.296ι	0±87.604ι	0±311.534ι	0±661.157ι
TB-FEM	0±29.276ι	0±87.616ι	0±311.554ι	0±661.315ι
SB-FEM	0±29.276ι	0±88.426ι	0±321.461ι	0±696.956ι
RB-FEM	0±29.324ι	0±88.495ι	0±328.188ι	0±739.088ι
EBB-FEM	0±29.325ι	0±89.357ι	0±341.356ι	0±805.312ι
$F_{D}=2\times 10^{5}$				
TB-AM	−27.687±9.572ι	−27.687±83.114ι	−27.687±310.301ι	−27.687±660.577ι
TB-FEM	−27.689±9.362ι	−27.689±83.277ι	−27.689±310.467ι	−27.689±660.854ι
SB-FEM	−27.739±9.362ι	−27.739±83.963ι	−27.739±320.262ι	−27.739±696.414ι
RB-FEM	−27.739±9.513ι	−27.739±84.209ι	−27.739±327.186ι	−27.739±738.720ι
EBB-FEM	−27.739±9.512ι	−27.739±84.943ι	−27.739±340.227ι	−27.739±804.834ι
$F_{D}=4\times 10^{5}$				
TB-AM	−8.383,-102.366	−55.375±67.883ι	−55.375±306.573ι	−55.375±658.834ι
TB-FEM	−8.353, −102.602ι	−55.376±68.630ι	−55.377±307.180ι	−55.377±659.468ι
SB-FEM	−8.353,1-02.603	−55.478±68.858ι	−55.475±316.638ι	−55.472±694.754ι
RB-FEM	−8.383,-102.572	−55.478±69.790ι	−55.478±324.159ι	−55.478±737.614ι
EBB-FEM	−8.383,-102.573	−55.478±70.049ι	−55.478±336.817ι	−55.478±803.399ι
$F_{D}=2\times 10^{8}$				
TB-AM	−55375.00, −0.015	−55374.9, −0.138	−55373.3, −1.752	−55367.1, −7.895
TB-FEM	−55416.00, −0.0154	−55380.2, −0.141	−55336.2, −1.86	−55222.5, −8.758
SB-FEM	−55493.8, −0.015	−55485.71, −0.140	−55471.27, −1.86	−55450.73, −8.758
RB-FEM	−55477.8, −0.015	−55477.8, −0.143	−55477.8, −2.100	−55477.8, −11.692
EBB-FEM	−55478.8, −0.015	−55478.8, −0.143	−55478.8, −2.100	−55478.8, −11.692

Note: TB-AM = Timoshenko Beam–Analytical Method; TB-FEM = Timoshenko Beam–Finite Element Method; SB-FEM = Shear Beam–Finite Element Method; RB-FEM = Rayleigh Beam–Finite Element Method; EBB-FEM = Euler–Bernoulli Beam–Finite Element Method.

**Table 4:** Damped and undamped frequencies of beam models supported a Filonenko-Borodich Foundation, considering $K_{r_{1}}=K_{r_{2}}=K_{l_{1}}=K_{l_{2}}=10^{7}$ and $K_{h}=q_{f}=10^{6}$.

BM	$\omega_{1}$	$\omega_{2}$	$\omega_{3}$	$\omega_{4}$
$F_{D}=0$				
TB-AM	0±32.009ι	0±89.756ι	0±313.350ι	0±662.752ι
TB-FEM	0±30.023ι	0±90.949ι	0±314.995ι	0±664.637ι
SB-FEM	0±30.023ι	0±91.789ι	0±324.992ι	0±700.422ι
RB-FEM	0±29.324ι	0±88.495ι	0±328.188ι	0±739.088ι
EBB-FEM	0±30.069ι	0±92.083ι	0±331.863ι	0±742.469ι
$F_{D}=2\times 10^{5}$				
TB-AM	−27.688±16.062ι	−27.688±85.379ι	−27.687±312.125ι	−27.687±662.174ι
TB-FEM	−27.738±11.486ι	−27.738±86.777ι	−27.738±313.919ι	−27.738±664.178ι
SB-FEM	−27.739±11.485ι	−27.739±87.497ι	−27.739±323.807ι	−27.739±699.873ι
RB-FEM	−27.738±11.606ι	−27.738±87.972ι	−27.738±330.871ι	−27.738±742.102ι
EBB-FEM	−27.739±11.606ι	−27.739±88.744ι	−27.739±344.052ι	−27.739±808.495ι
$F_{D}=4\times 10^{5}$				
TB-AM	−100.561,−10.188	−55.375±70.638ι	−55.375±308.419ι	−55.375±660.435ι
TB-FEM	−102.130, −8.825	−55.468±72.837ι	−55.468±310.668ι	−55.468±662.799ι
SB-FEM	−102.131, −8.825	−55.478±73.126ι	−55.475±320.223ι	−55.475±698.222ι
RB-FEM	−102.100,−8.855	−55.478±74.285ι	−55.478±327.879ι	−55.478±741.002ι
EBB-FEM	−102.101,−8.855	−55.478±74.613ι	−55.478±340.478ι	−55.478±807.066ι
$F_{D}=2\times 10^{8}$				
TB-AM	−55375.00, −0.018	−55374.9, −0.145	−55373.3, −1.773	−55367.1, −7.933
TB-FEM	−55478.8, −0.016	−55474.9, −0.151	−55463.2, −1.903	−55443.5, −8.8453
SB-FEM	−55493.8, −0.016	−55485.6, −0.151	−55471.2, −1.903	−55450.6, −8.845
RB-FEM	−55478.0, −0.016	−55478.0, −0.155	−55478.2,−2.147	−55478.0, −11.799
EBB-FEM	−55478.6, −0.0162	−55478.6, −0.1558	−55476.6, −2.147	−55467.0, −11.7986

Note: TB-AM = Timoshenko Beam–Analytical Method; TB-FEM = Timoshenko Beam–Finite Element Method; SB-FEM = Shear Beam–Finite Element Method; RB-FEM = Rayleigh Beam–Finite Element Method; EBB-FEM = Euler–Bernoulli Beam–Finite Element Method.

**Fig 15:**
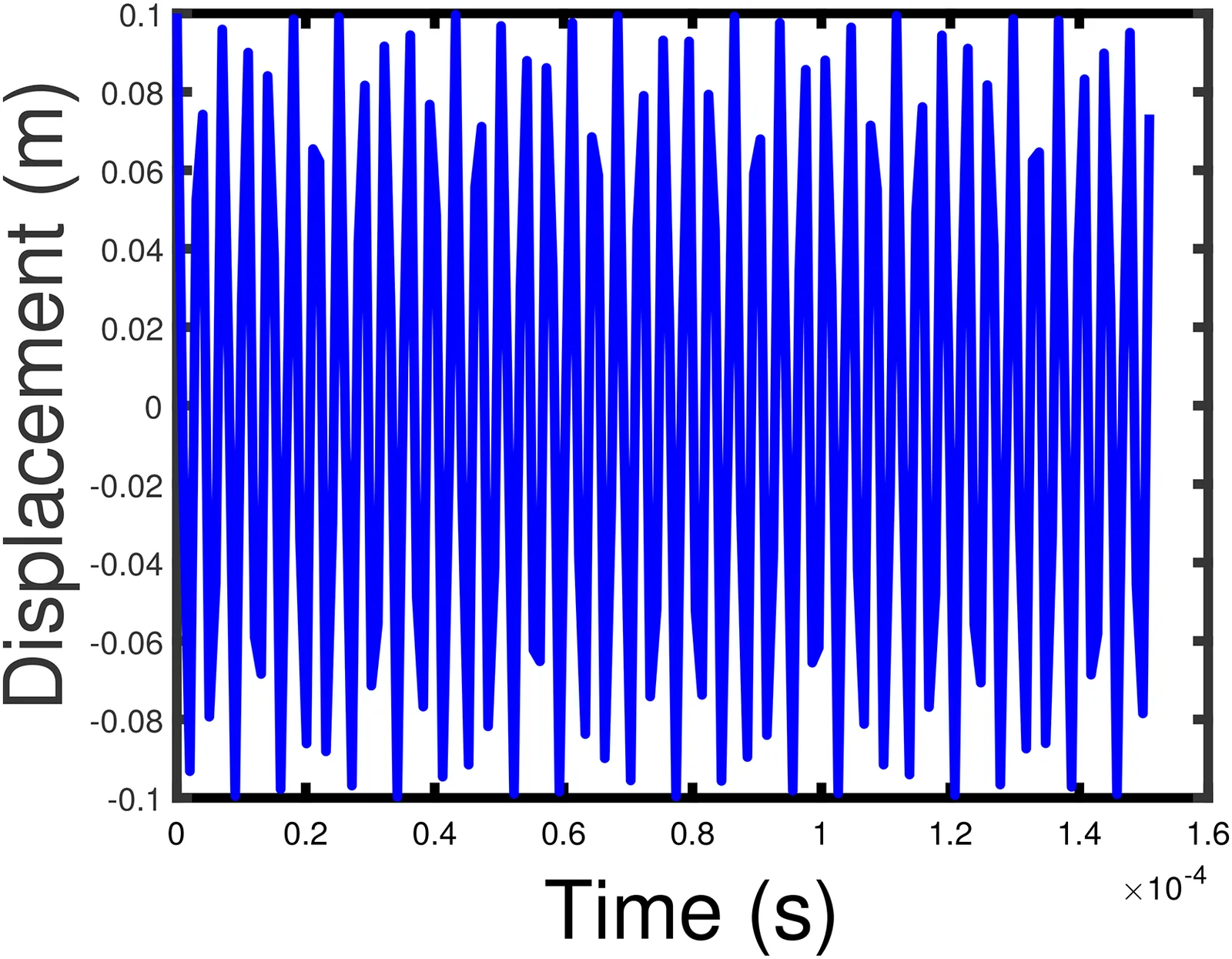
Oscillatory response of the TB over the Filonenko–Borodich Foundation for given $K_{l_{1}}=K_{l_{2}}=10^{13}$, $K_{r_{1}}=K_{r_{2}}=0$, and $K_{h}=10^{5}$, with different values of $F_{D}=0$.

**Fig 16:**
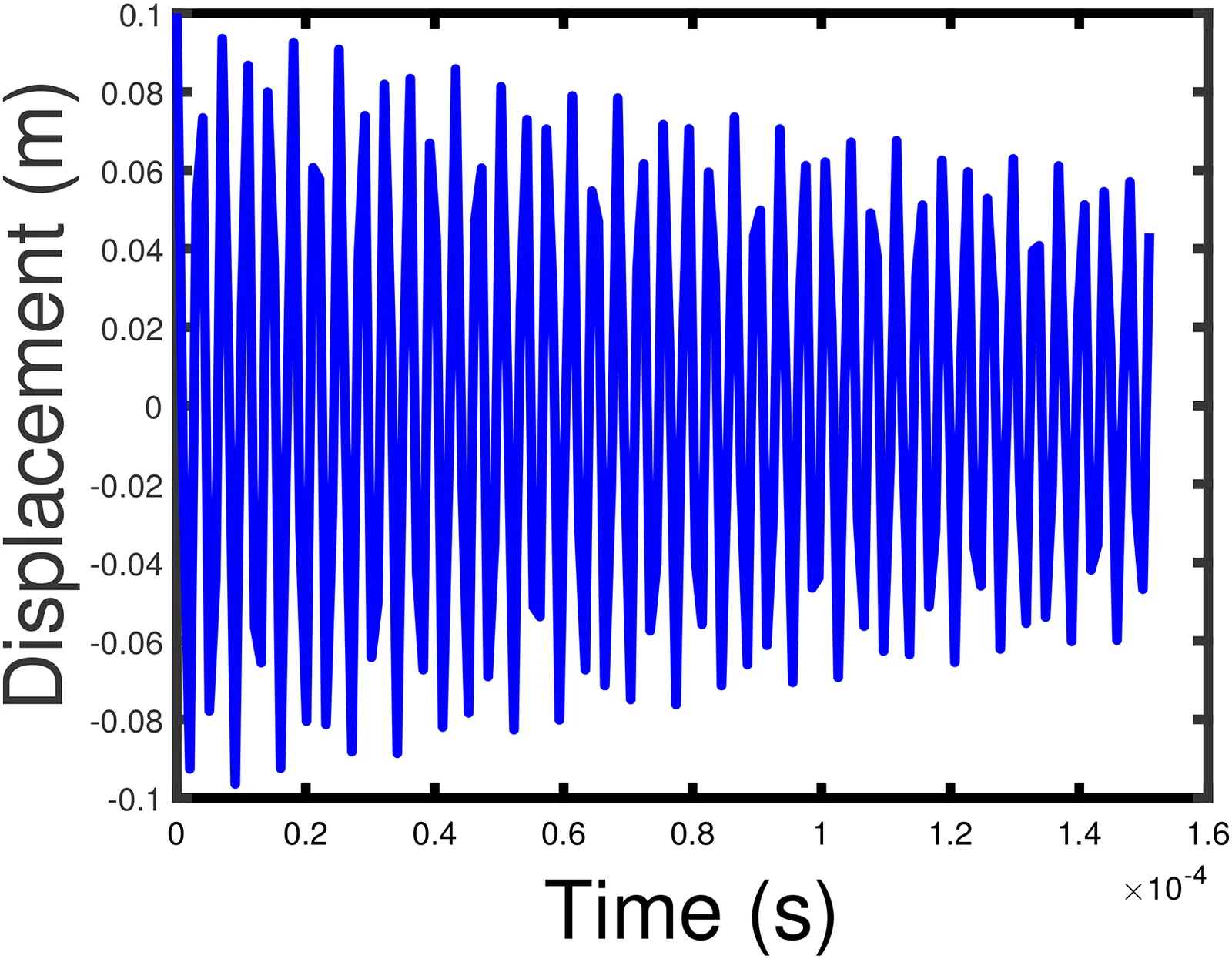
Oscillatory response of the TB over the Filonenko–Borodich Foundation for given $K_{l_{1}}=K_{l_{2}}=10^{13}$, $K_{r_{1}}=K_{r_{2}}=0$, and $K_{h}=10^{5}$, with different values of $F_{D}=2\times 10^{7}$.

**Fig 17:**
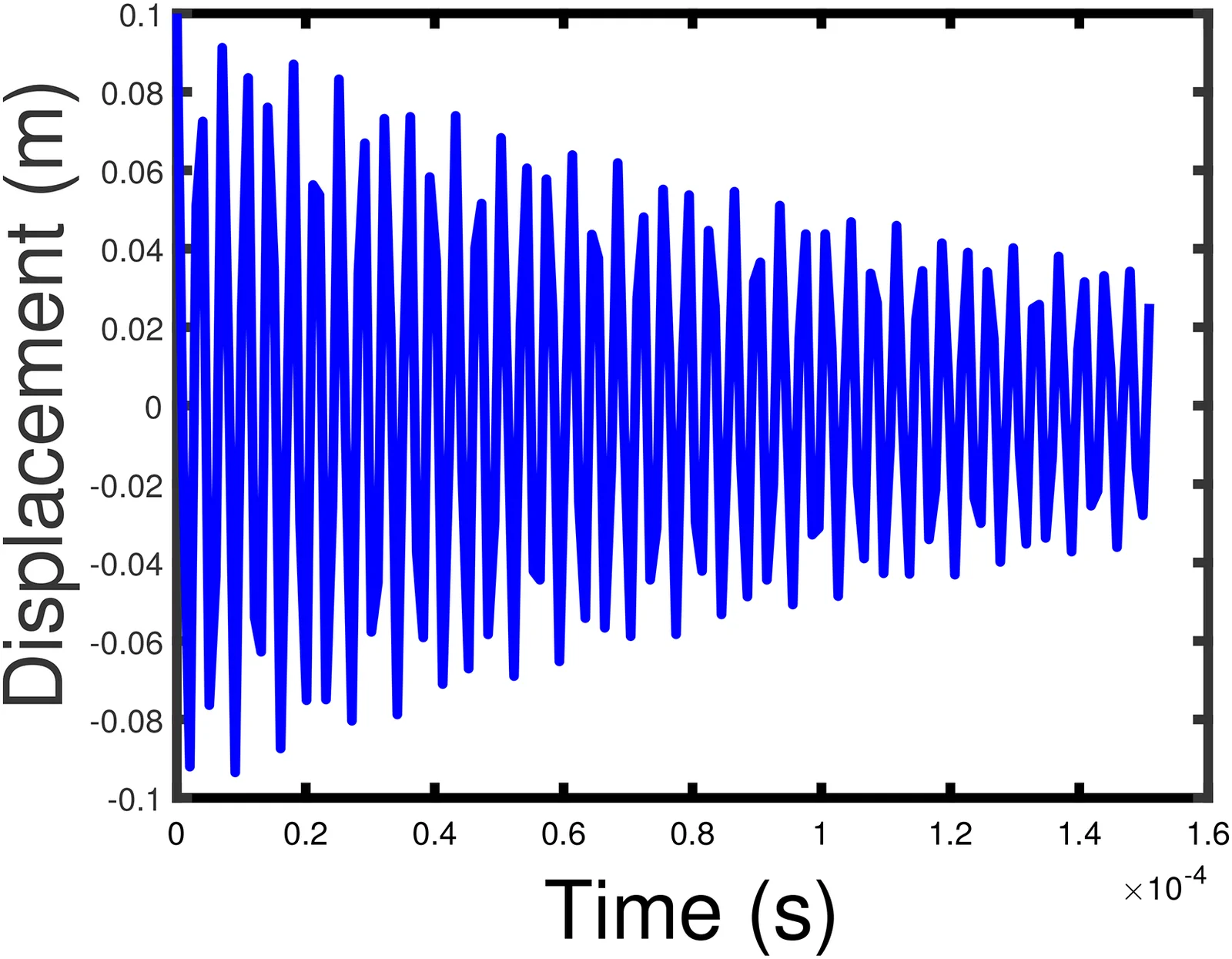
Oscillatory response of the TB over the Filonenko–Borodich Foundation for given $K_{l_{1}}=K_{l_{2}}=10^{13}$, $K_{r_{1}}=K_{r_{2}}=0$, and $K_{h}=10^{5}$, with different values of $F_{D}=4\times 10^{7}$.

**Fig 18:**
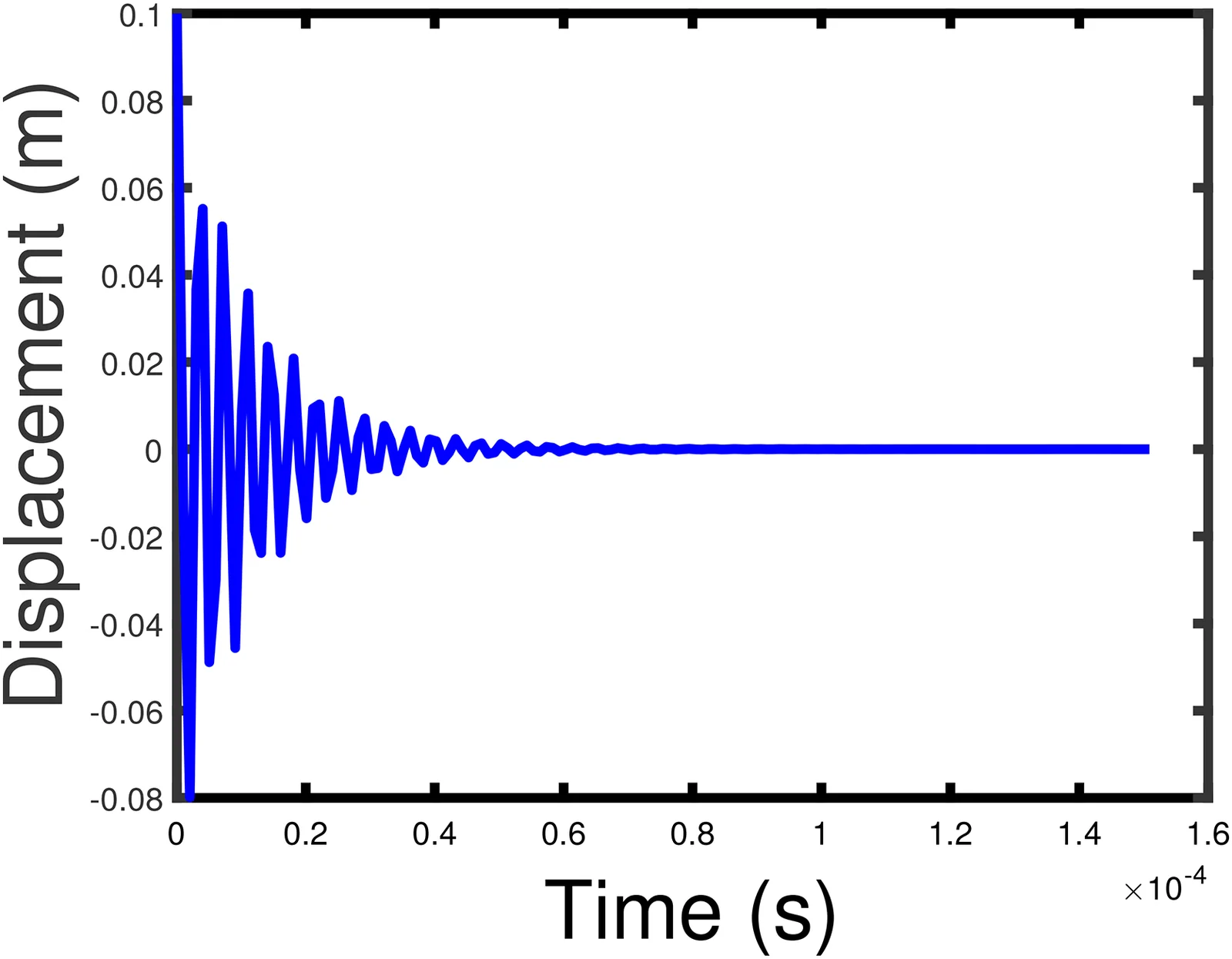
Oscillatory response of the TB over the Filonenko–Borodich Foundation for given $K_{l_{1}}=K_{l_{2}}=10^{13}$, $K_{r_{1}}=K_{r_{2}}=0$, and $K_{h}=10^{5}$, with different values of $F_{D}=1\times 10^{9}$.

**Fig 19:**
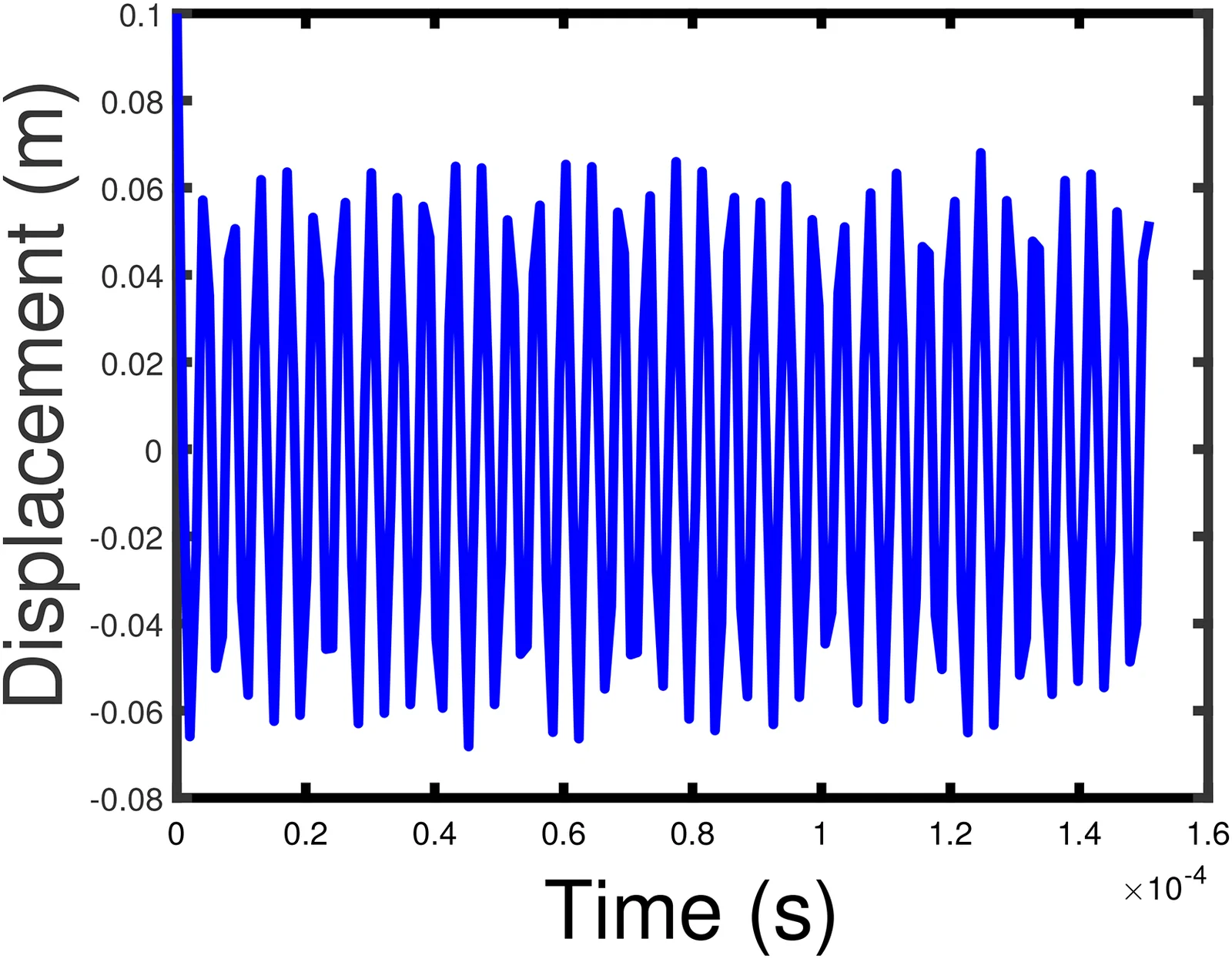
Oscillatory response of the SB over the Filonenko–Borodich Foundation for given $K_{l_{1}}=K_{l_{2}}=10^{13}$, $K_{r_{1}}=K_{r_{2}}=10^{13}$, and $K_{h}=10^{5}$, with different values of $F_{D}=0$.

**Fig 20:**
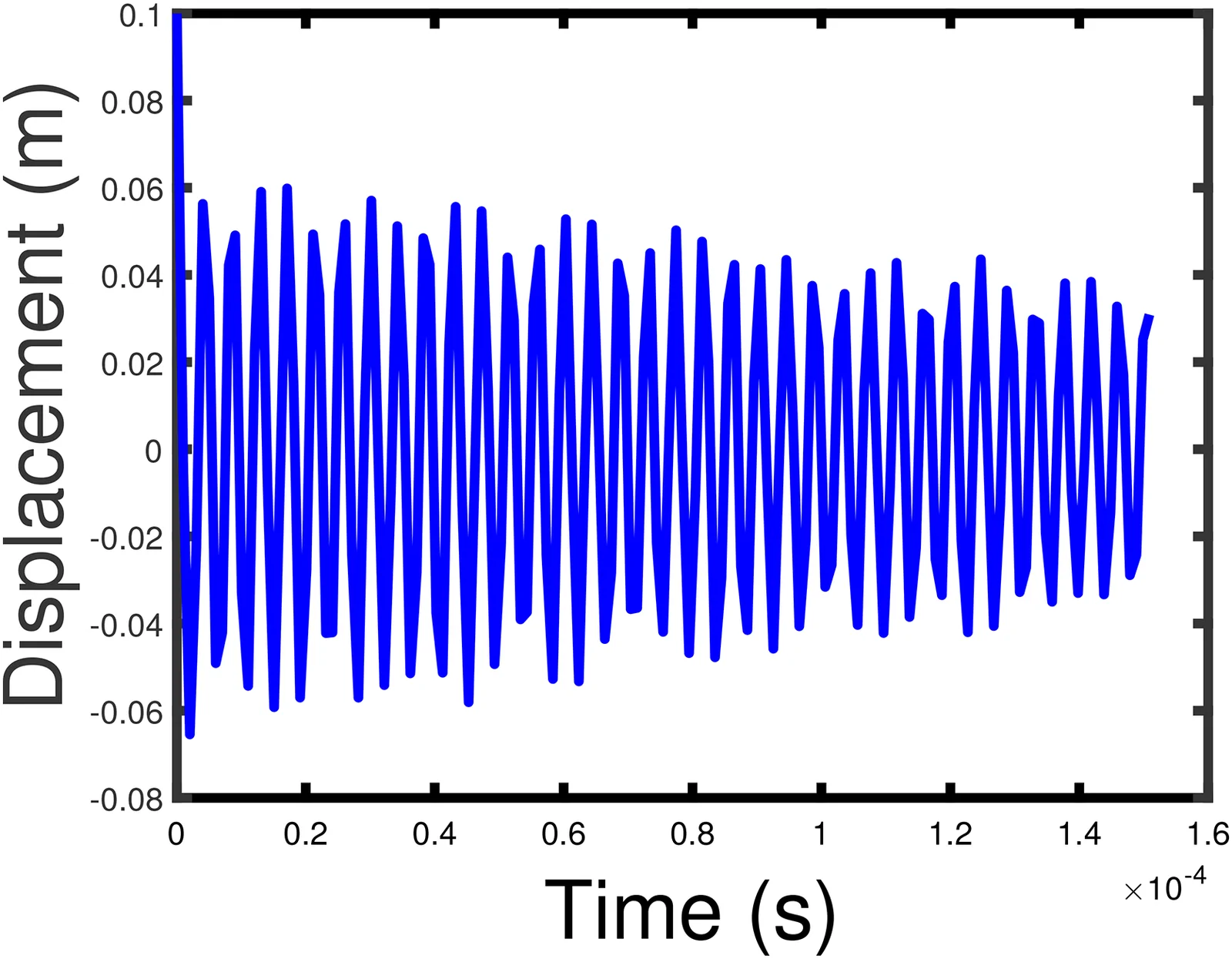
Oscillatory response of the SB over the Filonenko–Borodich Foundation for given $K_{l_{1}}=K_{l_{2}}=10^{13}$, $K_{r_{1}}=K_{r_{2}}=10^{13}$, and $K_{h}=10^{5}$, with different values of $F_{D}=4\times 10^{6}$.

**Fig 21:**
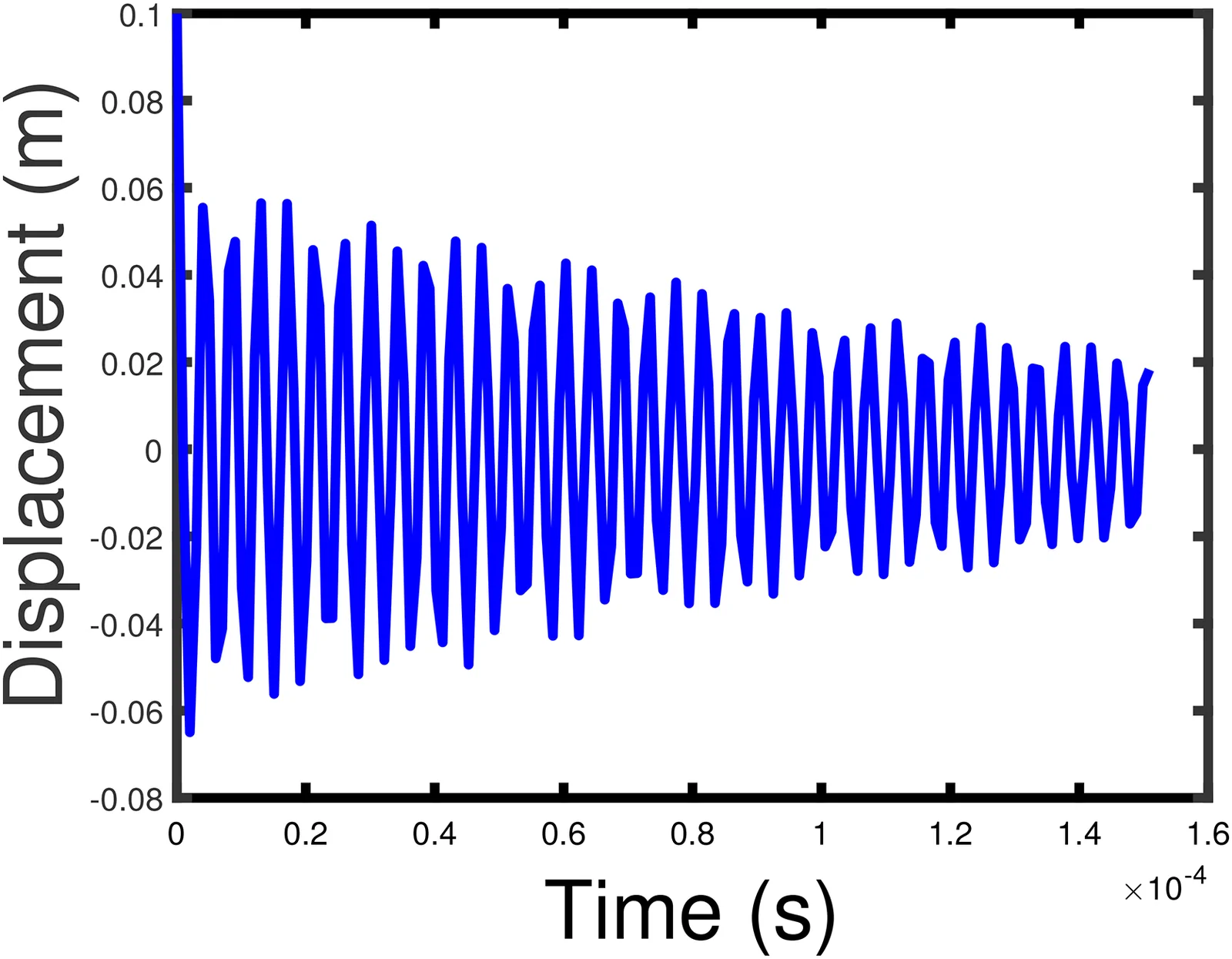
Oscillatory response of the SB over the Filonenko–Borodich Foundation for given $K_{l_{1}}=K_{l_{2}}=10^{13}$, $K_{r_{1}}=K_{r_{2}}=10^{13}$, and $K_{h}=10^{5}$, with different values of $F_{D}=8\times 10^{6}$.

**Fig 22:**
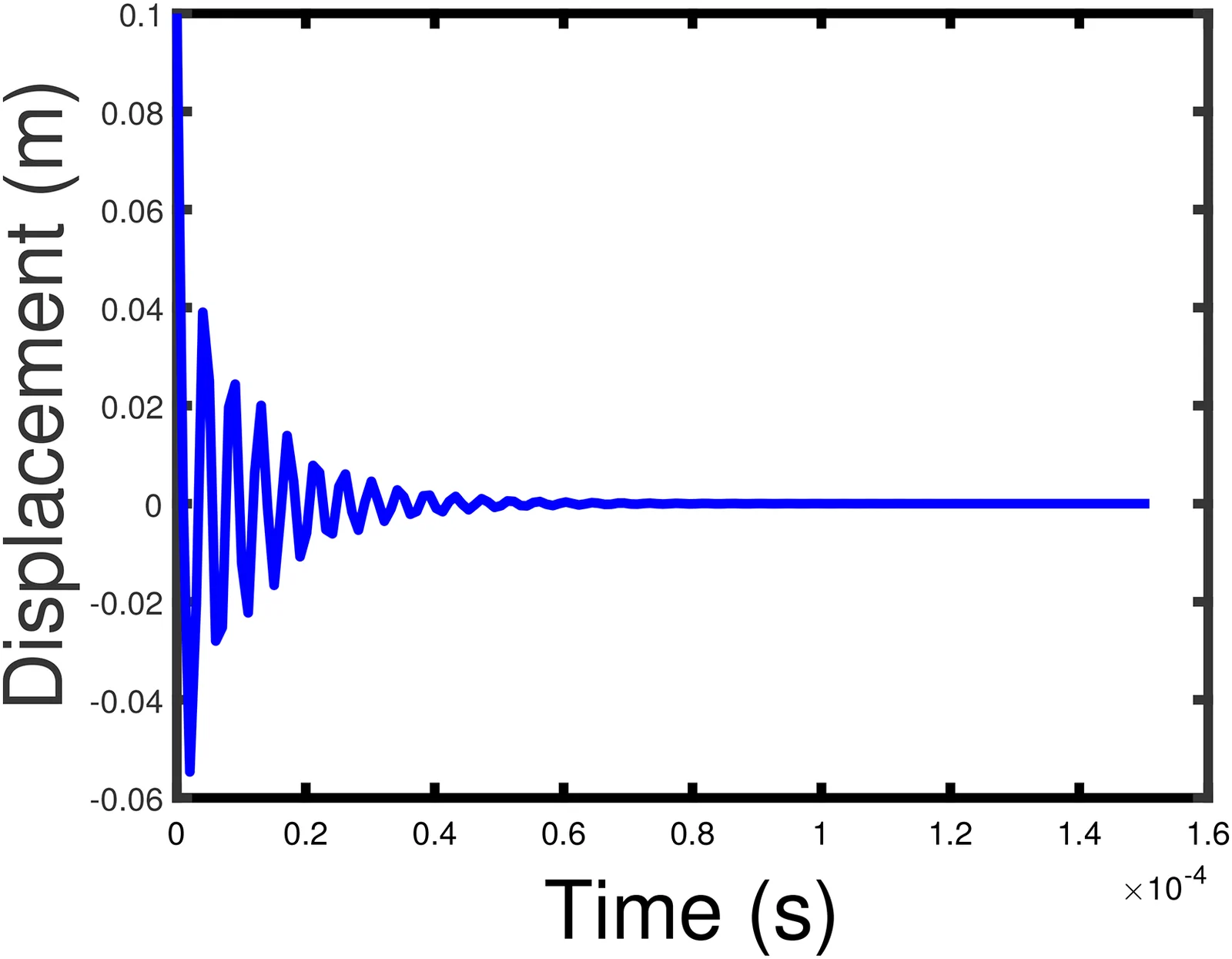
Oscillatory response of the SB over the Filonenko–Borodich Foundation for given $K_{l_{1}}=K_{l_{2}}=10^{13}$, $K_{r_{1}}=K_{r_{2}}=10^{13}$, and $K_{h}=10^{5}$, with different values of $F_{D}=1\times 10^{8}$.

**Fig 23:**
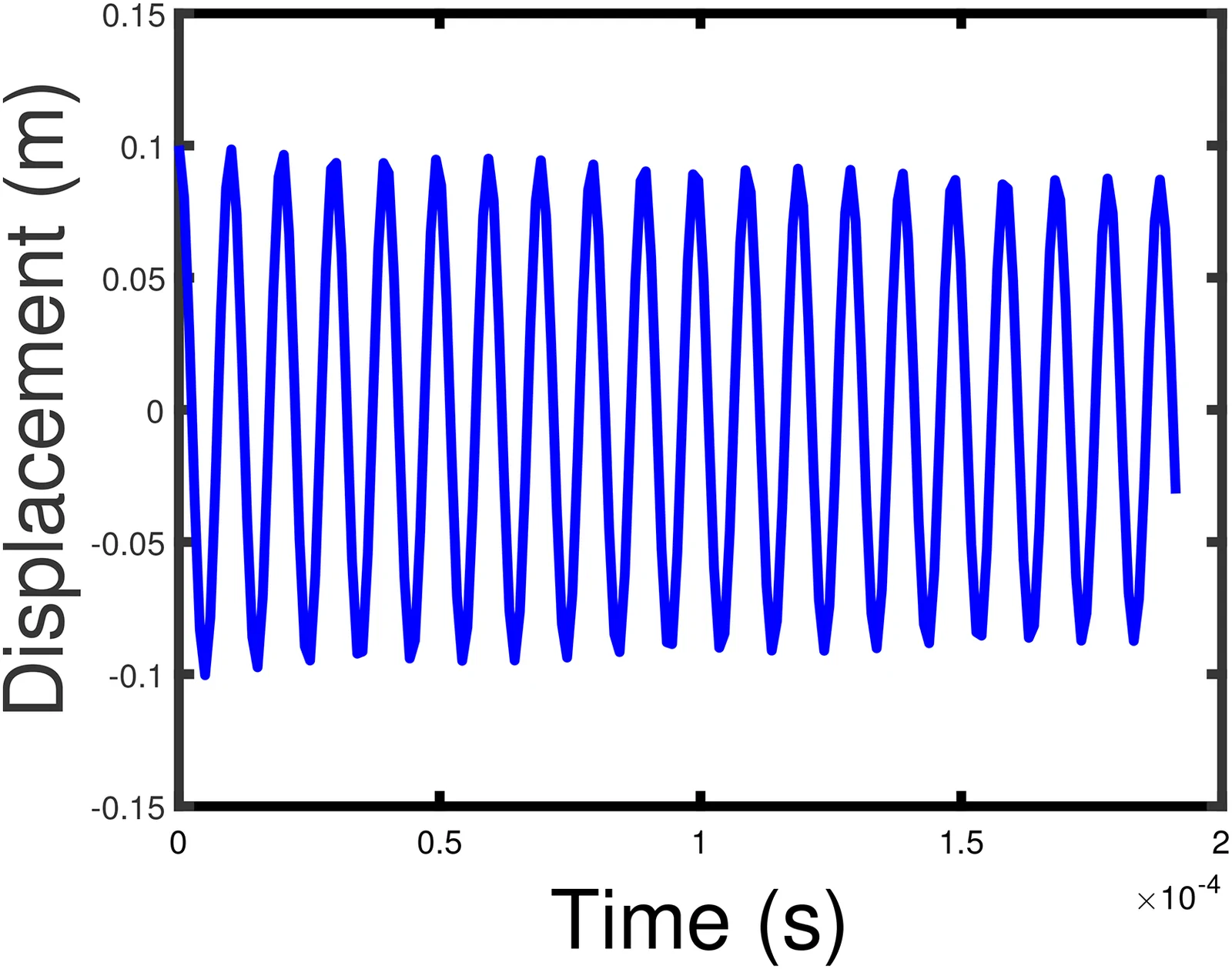
Oscillatory response of the RB over the Filonenko–Borodich Foundation for given $K_{l_{1}}=K_{l_{2}}=10^{13}$, $K_{r_{1}}=K_{r_{2}}=10^{13}$, and $K_{h}=10^{5}$, with different values of $F_{D}=0$.

**Fig 24:**
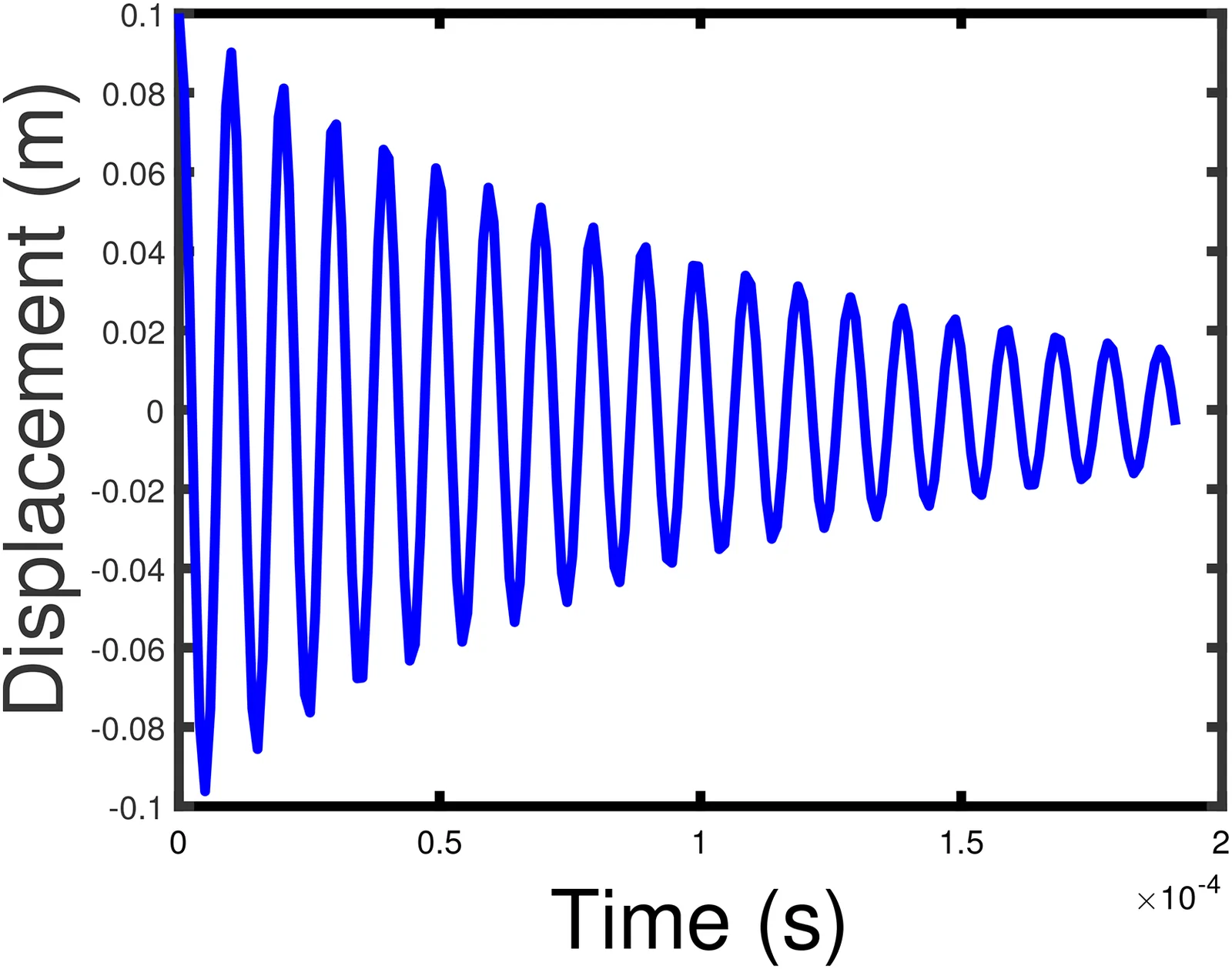
Oscillatory response of the RB over the Filonenko–Borodich Foundation for given $K_{l_{1}}=K_{l_{2}}=10^{13}$, $K_{r_{1}}=K_{r_{2}}=10^{13}$, and $K_{h}=10^{5}$, with different values of $F_{D}=2\times 10^{7}$.

**Fig 25:**
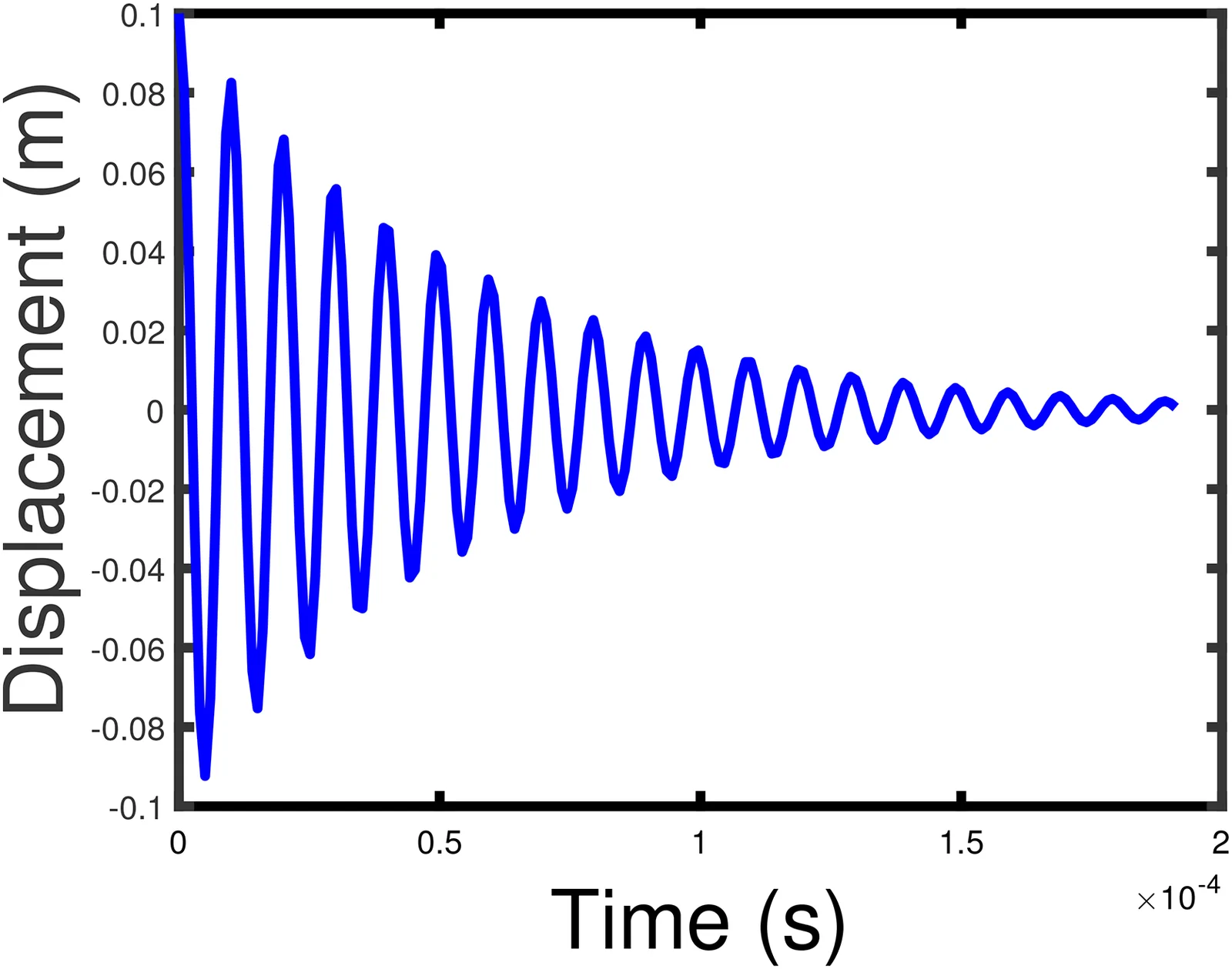
Oscillatory response of the RB over the Filonenko–Borodich Foundation for given $K_{l_{1}}=K_{l_{2}}=10^{13}$, $K_{r_{1}}=K_{r_{2}}=10^{13}$, and $K_{h}=10^{5}$, with different values of $F_{D}=4\times 10^{7}$.

**Fig 26:**
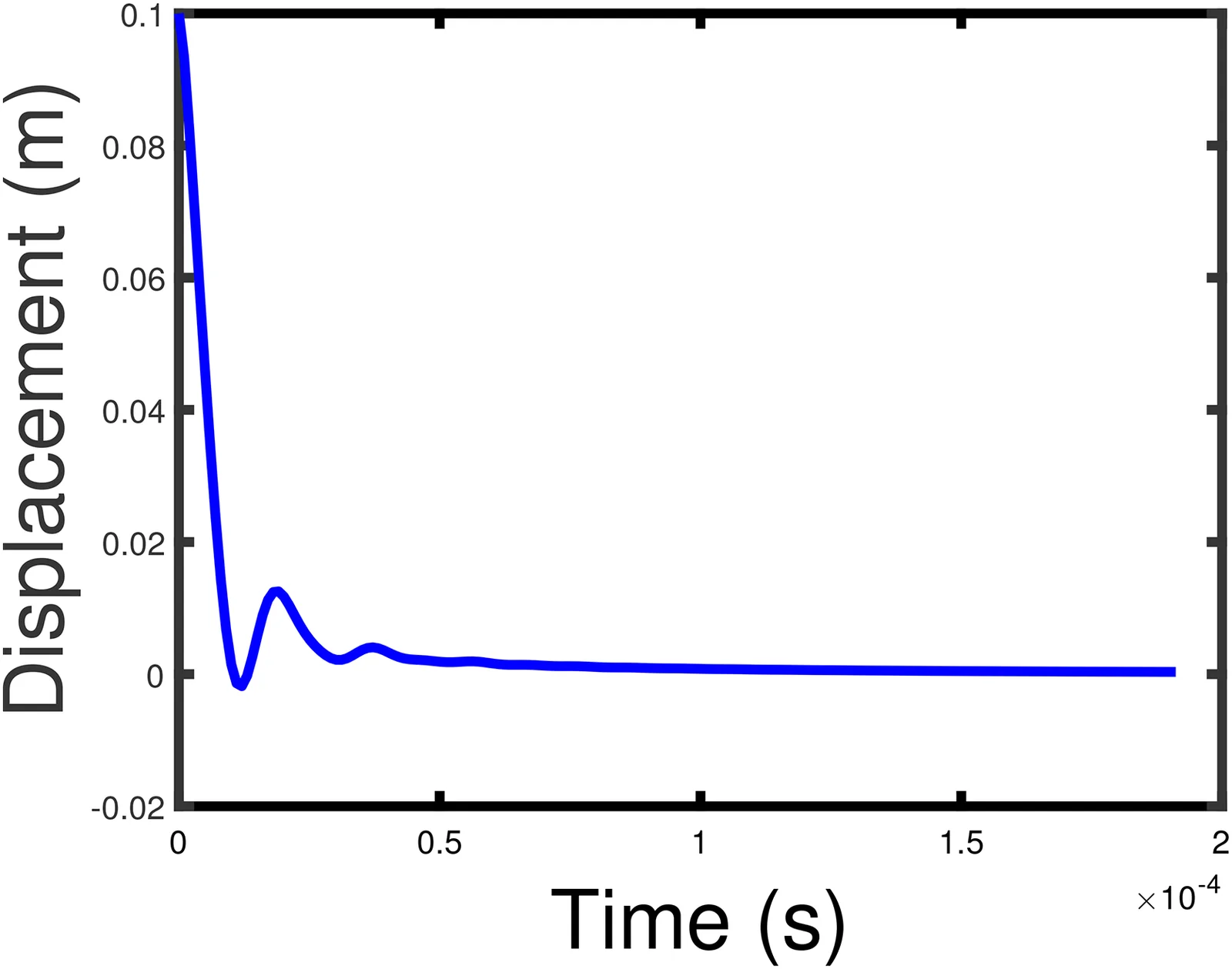
Oscillatory response of the RB over the Filonenko–Borodich Foundation for given $K_{l_{1}}=K_{l_{2}}=10^{13}$, $K_{r_{1}}=K_{r_{2}}=10^{13}$, and $K_{h}=10^{5}$, with different values of $F_{D}=1\times 10^{10}$.

Table 5 displays the undamped eigenfrequencies of different beam models under various boundary conditions. This detailed investigation of the effects of foundation types on the vibrational properties of undamped beams provides great insight into their dynamic responsiveness. The study demonstrates how the interaction of foundation stiffness and structural factors has a significant impact on the frequency response. The results are consistent with those published in [38], indicating the precision and accuracy of the existing methodology. The additional restoring resistance from the elastic foundation, which limits the transverse deflection and raises the system stiffness, is responsible for the increase in natural frequency with foundation stiffness. The effective stiffness and inertia of the beam are altered by shear deformation and rotary inertia, which have a greater impact in the higher modes. Physically, the energy dissipation is boosted by raising the damping coefficient. As a result, the vibration amplitude decays more quickly. When the damping is strong enough, the dissipative impact outweighs the restoring and inertial forces, resulting in an overdamped response without persistent oscillations.

**Table 5:** Eigenfrequencies of distinct beams under various boundary conditions.

B.C	$K_{r_{1}}=K_{r_{2}}=K_{l_{1}}=K_{l_{2}}=10^{7}$ and $K_{h},q_{f}=10^{5}$.			
	$\omega_{1}$	$\omega_{2}$	$\omega_{3}$	$\omega_{4}$
TB-AM	32.01	89.76	313.35	662.75
TB-FEM	30.02	90.95	314.90	664.64
SB-FEM	30.02	91.79	324.90	700.42
RB-FEM	29.32	88.40	328.19	739.09
EBB-FEM	30.07	92.08	331.86	742.47
B.C	$K_{l_{1}}=K_{r_{2}}=10^{12},\;K_{l_{2}}=K_{r_{1}}=0,q_{f}=K_{h}=10^{4}$			
TB–AM	107.91	393.66	787.50	1237.62
TB–FEM	107.91	393.69	787.82	1238.90
[38]	107.92	393.60	787.82	1238.90
B.C	$K_{l_{1}}=K_{r_{1}}=10^{10},\;K_{l_{2}}=K_{r_{2}}=10^{4},q_{f}=0,K_{h}=10^{4}$			
TB–AM	39.16	220.97	543.68	924.34
TB–FEM	39.16	220.97	543.46	924.34
[38]	39.17	220.98	543.46	924.34
BC	$K_{r_{1}}=K_{r_{2}}=0$, $K_{l_{1}}=K_{l_{2}}=10^{13}$, $K_{h}=10^{5}$ and $q_{f}=EI$			
TB-AM	113.56	398.03	789.63	1236.48
TB-FEM	114.75	389.78	795.23	1246.87
SB-FEM	115.60	410.53	824.30	1299.38
RB-FEM	118.21	439.16	939.33	1579.04
EBB-FEM	119.31	455.12	1014.59	1797.61

## Conclusion

The researchers investigated the vibrational behavior of Timoshenko, shear, Rayleigh, and Euler-Bernoulli beams supported by viscoelastic Filonenko-Borodich and Hetényi foundation models. This work provides important information regarding how changes in damping value, shear deformation, foundation stiffness, and rotary inertia affect the free vibrational properties of these beams, taking into account both undamped and damped scenarios. The significant discoveries include the following:

- Several elastic foundation models are used to explore the dynamic behavior of shear, Timoshenko, Rayleigh, and Euler-Bernoulli beams using the Galerkin finite element method and the method of separation of variables.
- The research builds on past work by incorporating non-classical boundary conditions, resulting in more realistic and adaptable modeling scenarios.
- It has been discovered that firmer foundations provide higher resistance to deflection and improve vibration control by increasing the natural frequencies of the beams.
- This study methodically shows how the damping coefficient impacts the system’s dynamic response, from undamped harmonic oscillations to underdamped vibrations with progressive amplitude decay to overdamped non-oscillatory exponential decay as damping magnitude increases.
- The finite element approach findings correspond closely with analytical solutions, demonstrating numerical precision and efficient convergence.
- Elastic foundations have a significant impact on vibration behavior, with each foundation’s shear layer and stiffness directly influencing a system’s dynamic behavior.
- This work investigated and examined the interplay of damping, shear effects, rotating inertia, and elastic foundations. The findings have applications in beam design and structural systems that need careful consideration of damping, shear effects, rotating inertia, and elastic foundations.
- The real part of the Timoshenko beam eigenvalue increases from −27.687 to −217.618 when the damping coefficient $F_{D}$ is increased from $2\times 10^{5}$ to 10^6^ Ns/m, while the imaginary (oscillatory) part reduces correspondingly. The change from underdamped to more severely damped motion is clearly indicated by this numerical signature. The system reaches full over damping (real, non-oscillatory eigenvalues) at $F_{D}=10^{9}$ Ns/m.
- A stiffer viscoelastic foundation is advantageous for a beam’s deflection resistance and passive vibration control; for instance, the fundamental Timoshenko beam frequency increases from 113.56 rad/s to 113.80 rad/s as $K_{h}$ increases from 10^4^ to 10^6^ N/m^2^ over the Hetényi foundation. Increasing the foundation stiffness $K_{h}$ increases the natural frequencies for all four beam models without changing the qualitative vibration pattern.

The current study considerably improves our understanding of beam response by investigating beam behavior on viscoelastic foundations. The state-space formulation in the form of Eq. (49) gives a systematic and computationally convenient representation of the governing equations, allowing for easy application of the proposed model to standard numerical time-integration schemes, e.g., the fourth-order Runge–Kutta method. The present vibration model is formulated in such a way that it can be numerically implemented and extended for practical engineering applications with different loading, damping, foundation and boundary conditions. Future study could incorporate non-uniform and tapered cross-sections, variable material characteristics, and spatially dispersed damping into the suggested model. Furthermore, studying how external elements like temperature and moisture variations affect the dynamic response could provide useful insights for building more durable and practical structures.
